# Fabrication of hydrogel mini-capsules as carrier systems

**DOI:** 10.12688/openreseurope.16723.2

**Published:** 2024-10-25

**Authors:** Elisa Roberti, Gaia Petrucci, Francesco Bianciardi, Stefano Palagi

**Affiliations:** 1 The BioRobotics Institute, Sant'Anna School of Advanced Studies, Pisa, Tuscany, 56025, Italy; 2 Department of Excellence in Robotics & AI, Sant'Anna School of Advanced Studies, Pisa, Tuscany, 56127, Italy

**Keywords:** alginate, agarose, core-shell, hydrogel capsules, microrobotics

## Abstract

Conventional drug administration often results in systemic action, thus needing high dosages and leading to potentially pronounced side effects. Targeted delivery, employing carriers like nanoparticles, aims to release drugs at a target site, but only a small fraction of nanoparticles actually reaches it. Microrobots have been proposed to overcome this issue since they can be guided to hard-to-reach sites and locally deliver payloads. To enhance their functionality, we propose microrobots made as deformable capsules with hydrogel shells and aqueous cores, having the potential added advantages of biocompatibility, permeability, and stimulus-responsiveness. Endowing microrobots with deformability could allow them to navigate inside capillaries and cross barriers to finally reach the target site. In this study, we present a cost-effective method for fabricating core-shell structures without the use of organic solvents, surfactants, or extreme pH conditions unlike other techniques (e.g. Layer by Layer). The process begins with the dripping of a mixture of hydrogels, agarose and alginate, into a solution to gelate the drops into beads. After they are loaded with calcium ions at different concentrations, they are immersed in an alginate solution to form the shell. Finally, the beads are heated to let the agarose melt and diffuse out, leaving a liquid core. By varying the concentration of calcium ions, we obtain shells of different thicknesses. To estimate it, we have developed a method using the colour intensity from microscope images. This allowed us to observe that lowering the calcium ions concentration below a threshold does not lead to the formation of continuous shells. For higher concentrations, although the core may remain partially gelled, continuous shells successfully form. Therefore, our fabrication process could find applications in drug delivery, encapsulation systems, and microrobotics.

## Plain language summary

Conventional drug administration methods often lead to widespread effects in the body and dosage-related side effects. Targeted drug delivery aims to release drugs precisely where they’re needed by using carriers. However, only a few of them actually reach the intended target. To address this issue, scientists are exploring microrobots, tiny machines that can reach places deep inside the body. We design microrobots as soft liquid-filled capsules. In our research, we’ve developed an affordable method to make these capsules without using harsh chemicals. The obtained capsules have a controllable shell thickness and a liquid or almost liquid core. This makes our capsules promising candidates as microrobot bodies or as drug delivery carriers.

## 1 Introduction

Most routes of drug administration ( *e.g.* intravenous) lead to systematic action of the drug, as topical administration is possible only for suitable diseases. In many cases, this requires much higher dosages than those needed if the administration could be topical, resulting in much more significant side effects. This is particularly problematic for chemotherapy. To address this issue, conventional drug delivery approaches aim to improve aqueous solubility and chemical stability of the drug, increase its pharmacological activity and reduce side effects while maintaining the therapeutic concentrations at the target site. A suitable approach to reduce drugs’ side effects consists in adopting carriers, such as nanoparticles, that could release the drug only at the target site or to target tissues or cells, which is known as targeted drug delivery. Whereas nanoparticles-based targeted drug delivery has proved to enhance the therapeutic index and reduce adverse side effects, there are still open issues related to drugs toxicity, selectivity and dosage. One key problem is that only a tiny fraction of the drug-loaded nanoparticles reaches the target site (e.g. a solid tumour) ^1^.

Microrobots have been proposed to overcome these issues ^2^. The main feature of these tiny robotic devices is their controlled navigation, which could allow them to be guided to hard-to-reach target sites inside the human body. Microrobotics could thus become a non-invasive approach to topically administer drugs (or drug-loaded carriers) at sites that are not currently suited to topical administration. Although recent advances in microrobotics for drug delivery applications, tracking and guiding microrobots *in vivo* remains challenging, and their ability to move through body tissues and cross biological barriers is still limited ^2^. To make microrobots ultra-deformable and able of moving across tissues and barriers, a possible design consists of a thin, soft shell enveloping a liquid core ^3, 4^. Ultra-deformability refers to the ability of these microrobots to deform substantially and reversibly allowing them to pass through openings ^5– 8^. We take inspiration from leukocytes, particularly neutrophils and macrophages, which are sufficiently deformable to move inside small capillaries (< 5 µm) that reach solid tumours, travel through the interstitial spaces of tissues (< 1 µm), and extravasate through hundreds-nanometres junctions between cells of the leaky vasculature in the tumour microenvironment ^1^. Moreover, the microrobots’ building materials should be biocompatible or biodegradable. Additionally, the shell should be permeable and possibly stimuli-responsive, releasing.

Core-shell and hollow particles can be prepared using a variety of methods ^9, 10^, including: Layer-by-Layer (LbL) ^11, 12^, sol-gel ^13, 14^, solvent evaporation, spray-drying ^15^, double emulsions (O/W/O or W/O/W) ^16^. However, none of these methods is suited to the fabrication of the ultra-deformable microrobots we have devised, which requires mild process conditions to avoid damages to the potential payloads.

In this article, we introduce a method for fabricating core-shell capsules that have a liquid aqueous core and a hydrogel shell, which involves no organic solvents, surfactants, or low/high pHs. We exploit the different gelation mechanisms of alginate and agarose to create the core-shell capsules. Specifically, alginate crosslinks in the presence of calcium ions, while agarose gelate at room temperature. We report the characterization of the obtained core-shell capsules, with a focus on the shell thickness and its dependence on the process parameters. In particular, obtaining a thin yet continuous shell is essential for the capsules to be deformable yet stable. The proposed process holds potential also for the fabrication of micro-capsules and core-shell particles for different applications, including drug-delivery systems other than microrobotics ones.

## 2 Methods

### 2.1 Materials

Sodium alginate (CAS 9005-38-3), agarose (CAS 9012-36-6), sodium citrate dihydrate (SCD)(CAS 6132-04-3), calcium chloride (CAS 10043-52-4) and iron (III) oxide nanopowder <50 nm (CAS 1309-37-1) were purchased from Merck. MilliQ water is used unless differently stated (Elix ^®^ Advantage 10 Water Purification System).

To fabricate the shell we have chosen alginate, a natural polymer which shows attractive properties such as biocompatibility, low cost, ease of gelation, inert nature ^17^. Alginate undergoes a reversible gelation process when divalent ions ( *e.g.* Ca ^2+^, Ba ^2+^ or Sr ^2+^) are added to the polymeric solution and it returns to the original liquid form when exposed to chelating agents that bind the divalent ions with a higher affinity constant ^18^.

### 2.2 Preparation of the core-shell particles

**Figure 1.**
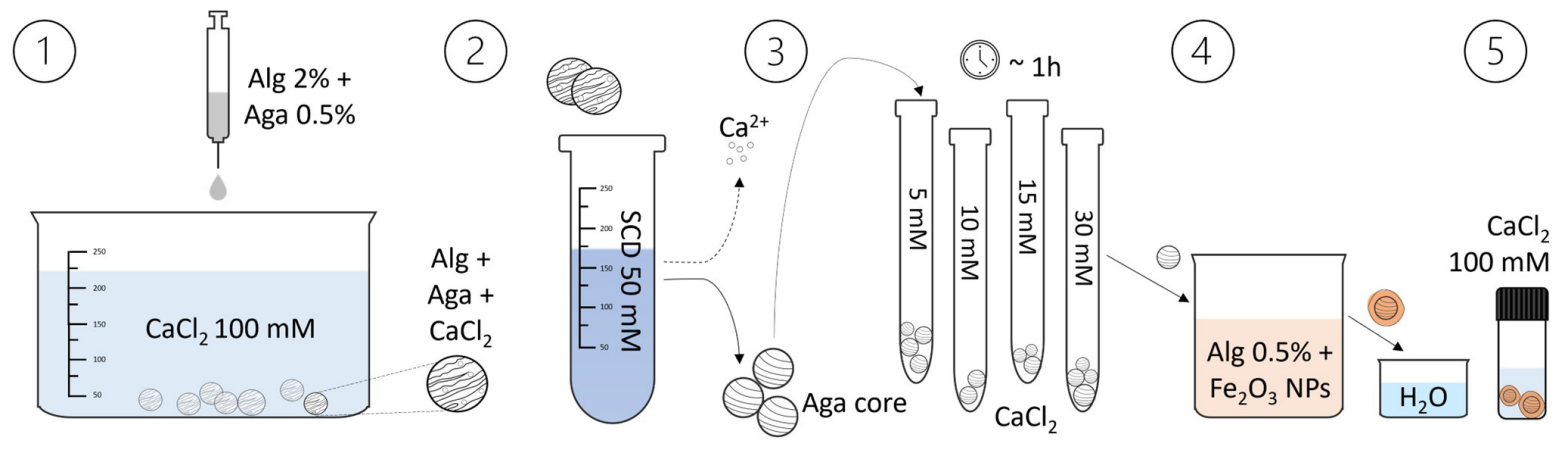
Illustration of the core-shell particles fabrication process. The core-shell beads preparation involves the dripping of an agarose-alginate mixture in a CaCl _2_ solution, followed by washing and transferring the beads to a sodium citrate dihydrate (SCD) solution. After loading them with Ca ^2+^ ions (by equilibration in CaCl _2_ solutions of different concentrations), the beads are placed in an alginate solution containing Fe _2_O _3_ nanoparticles to form the shell. Subsequently, they are washed and transferred to another CaCl _2_ solution to consolidate the shell. By heating the beads, the agarose liquefies and diffuses out, resulting in a liquid core within the shell.

The process for preparing the core-shell beads is depicted in Figure 1 and a tutorial video can be found in the Extended Data ^19^.

1. The agarose-alginate water solution is prepared mixing a 1% w/v agarose solution (previously heated at 95°C to promote dissolution) and a 4% w/v alginate solution to have overall concentrations of 0.5% agarose and 2% alginate. The obtained warm solution is added drop-wise to a CaCl _2_ 100 mM solution using a 10 mL syringe (Terumo ^®^) to form gel beads because of the immediate crosslinking of alginate by calcium ions. The beads are kept in the CaCl _2_ solution for about 10 minutes to complete the crosslinking.
2. The beads are then washed with 100 ml of water to remove the excess of CaCl _2_, transferred into a 50 mM solution of sodium citrate dihydrate (SCD) and kept overnight. SCD is meant to chelate Ca ^2+^ ions and thus de-crosslink the alginate template, leaving beads of just agarose.
3. The agarose beads are then washed again with water and transferred in CaCl _2_ solutions at different concentrations (ranging from 0.1 mM to 100 mM).
4. After 1 hour of equilibration in the CaCl _2_ solution, the beads are picked up and briefly placed on a filter paper to remove the excess solution from the surface. They are then immersed for 15 minutes in a solution of alginate 0.5% w/v to form an alginate shell around the agarose bead templates. The alginate solution contains 0.2 mg/ml of 50 nm Fe _2_O _3_ nanoparticles to enhance the shell visualization under the optical microscope.
5. After 15 minutes, the core-shell structures are briefly rinsed in water and then transferred in a 100 mM CaCl _2_ solution to complete the crosslinking of the alginate shell.
6. To obtain a liquid core, the core-shell structures are placed in a thermal bath at a temperature of 90/95°C for 2–7 hours, allowing the agarose to liquefy and diffuse out, leading to microcapsules with an alginate shell and a liquid aqueous core.

We immersed capsules purposely made using cores loaded with particles (CaCO _3_ or SiO _2_) in a SCD solution to de-crosslink the shell and assess the core liquidity. The release of the particles from the capsules highlighted that the cores were insubstantial after 6–7 hours of thermal bath, although we still observed a light gel residue (see Extended Data, video “ChargedCapsuleSiO2_core”) ^19^.

### 2.3 Capsules characterization

***2.3.1 Shell formation and thickness***

We characterized the capsules using an Hirox optical digital microscope (Hirox HRX-01) and analysing the images by two different approaches: i) direct measure of the shell thickness – this method is however challenging for thin shells; ii) indirect measure based on shell colour.

**Figure 2.**
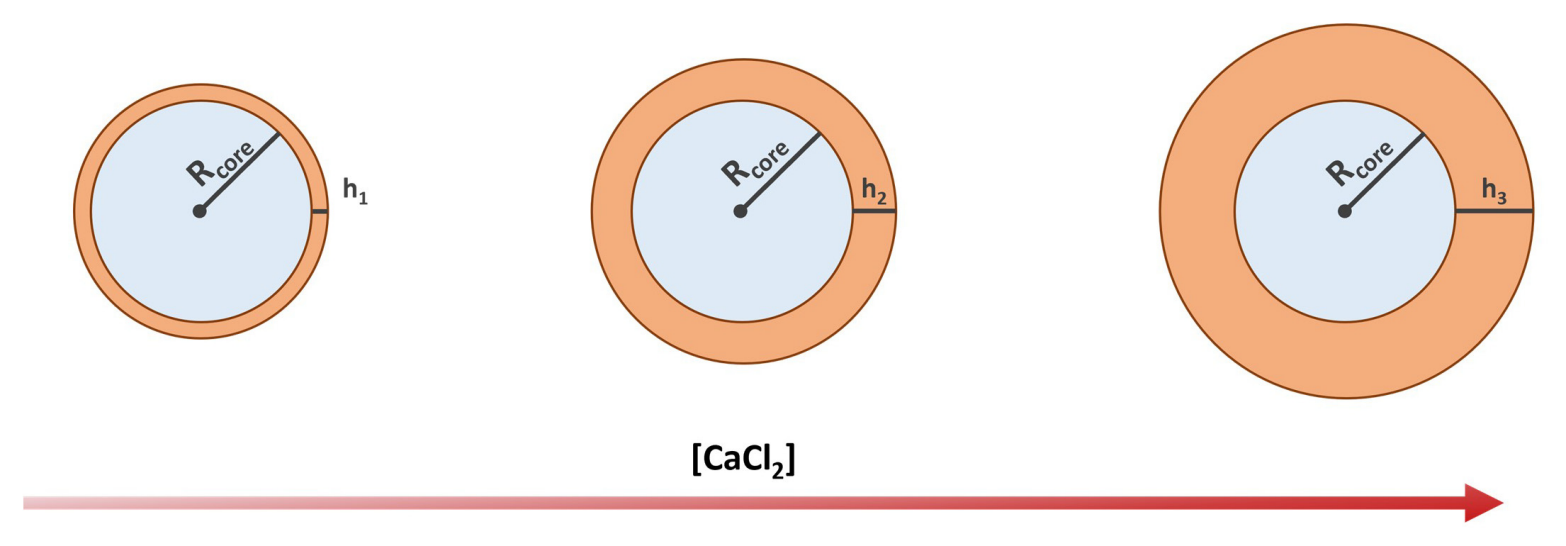
Schematics showing the expected shell thickness increase with the increasing concentration of calcium chloride.

We assume the volume *V _s_*, and thus the thickness of the alginate shell, to be proportional to the amount of Ca ^2+^ ions loaded into the core (see Figure 2), such that


$$\;V_{s}=\alpha \;n_{{\text{Ca}}^{\text{2+}}}\;(1)$$

where *α* is a proportionality constant (to be determined) and $n_{{\text{Ca}}^{\text{2+}}}=V_{c}[{\text{Ca}}^{2+}]$ is the number of Ca ^2+^ ions moles in the core ([Ca ^2+^] is assumed to be equal to the concentration of the CaCl _2_ solution the cores have been soaked in before forming the alginate shell). Considering that $V_{c}=\frac{4}{3}\pi R_{c}^{3}$ (where *R _c_* is the core radius) and $V_{s}=\frac{4}{3}\pi [{\left(R_{c}+h\right)}^{3}-R_{c}^{3}]$ (where is *h* the thickness of the shell), we obtain


$$h=R_{c}\left(\sqrt[{3}]{\alpha [{\text{Ca}}^{\text{2+}}]+1}-1\right)\;(2)$$

**Figure 3.**
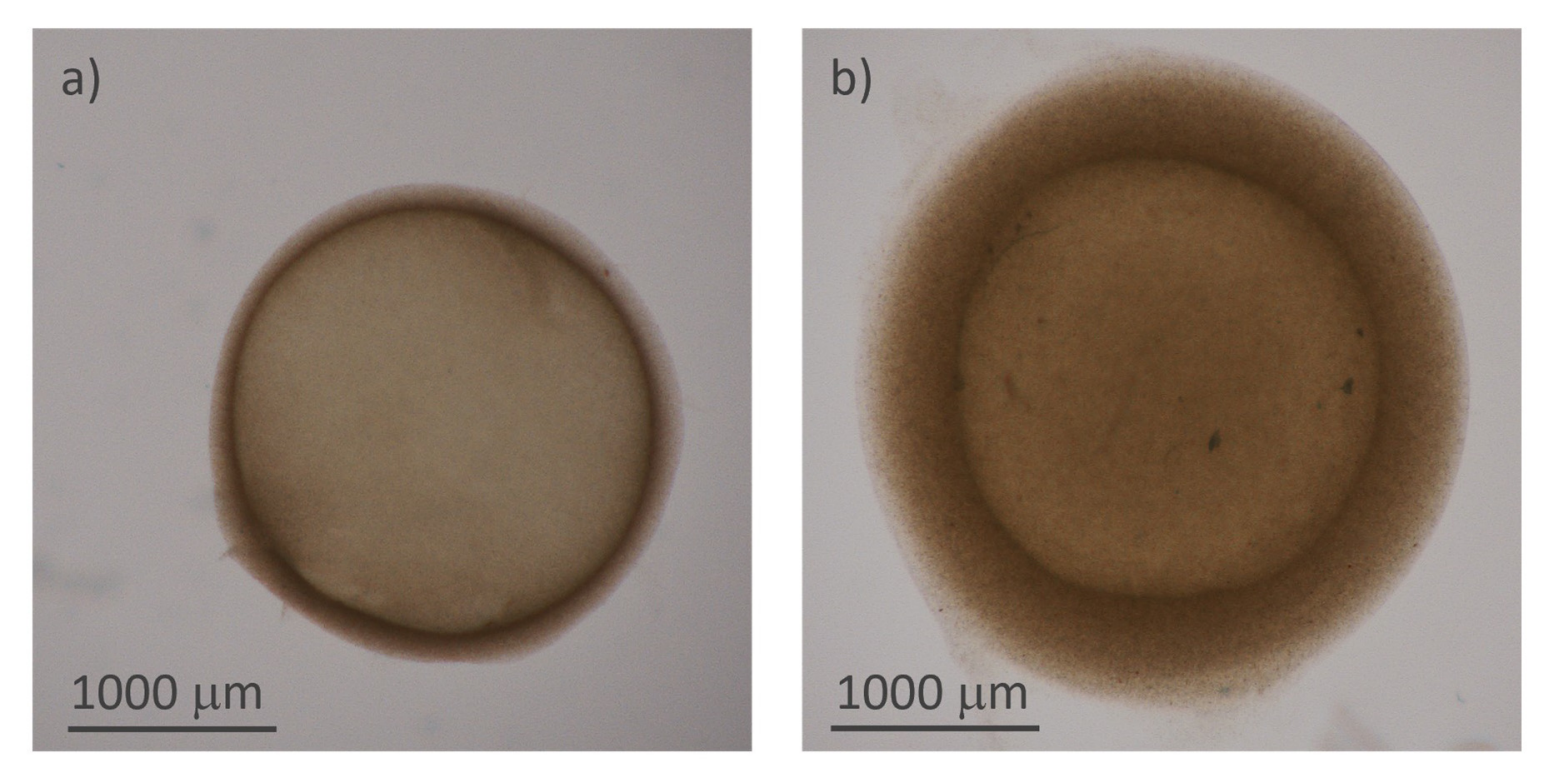
Structure of the hydrogel microcapsules: **a**) and **b**) optical microscope images of two capsules of different thicknesses

When the core is immersed in a solution at a fixed alginate concentration, the release of Ca ^2+^ ions induces the crosslinking of alginate around the cores. This produces a shell whose thickness depends on the number of Ca ^2+^ ions previously absorbed by the core. Therefore, we assume n Ca ^2+^ is and thus to *V _s_*. We then fabricated batches of capsules by soaking the agarose cores in CaCl _2_ solutions at concentrations ranging between 0.1 and 100 mM (0.1, 1, 2, 5, 10, 15, 20, 30, 50 and 100 mM, three to five samples for each concentration) – see Figure 3. We stained the shell by adding iron oxide nanoparticles to the alginate solution to enhance its detection and ease the measurement of its thickness (particles were chosen instead of a dye, as most dyes would diffuse away and/or stain the core too).

We first measured the shell thicknesses using the measuring tool of the digital optical microscope’s software. To perform a linear fit and obtain an estimate for *α*, we rearranged Equation 2 to obtain an equation of the form *y* = *ax*, leading to:


$${\left(\frac{h}{R_{c}}+1\right)}^{3}-1=\alpha [{\text{Ca}}^{\text{2+}}]\;(3)$$

Once obtained a value for *α*, Equation 2 can be used to calculate the expected shell thickness as a function of the CaCl _2_ solution concentration. Therefore, we estimated the thickness of the shells too thin to be directly measured by extrapolating values from the fitted curve.

To better estimate the thickness of such thin shells, we have devised a complementary approach based on the assumption that the shell colour intensity (given by the iron oxide nanoparticles) linearly depends on its thickness. To have consistent measurements of the colour intensity, we set and kept fixed the parameters for the microscope images acquisition (illumination, camera settings). The parameters values were chosen to obtain good quality images with all the inspected thicknesses. The image processing develops as follow:

1. each image of the set is segmented creating two clusters: one with all the pixels corresponding to the capsules, and a second one with the background;
2. in each image, a small portion at the centre of the capsule cluster is automatically selected, and the RGB values of the corresponding pixels are extracted;
3. in each image, an average value for each of the three colour channels is calculated over the selected region;
4. in each image, average channel values corresponding to the background are also calculated;
5. from the colour intensities extracted at point 3 and 4, the red channel value, the value of the red channel minus the background, and the value of the red channel minus the blue channel are calculated and related to the CaCl _2_ concentration and to the theoretical shell thickness (calculated with Equation 2);
6. a linear fit of the measured shell thickness over the measured R−B colour intensity is performed, obtaining a relation to estimate the shell thickness from the colour intensity.

From this we again extrapolate the expected thickness for the non-measurable shells and compare the predictions with those obtained from Equation 2.

***2.3.2 Core dissolution***

To achieve ultra-deformability, our core-shell capsules must consist of a highly soft shell and a predominantly liquid core, as previously mentioned. We assume that, due to the fabrication method, the core at step 6 of the protocol will melt and diffuse out of the alginate shell, leaving a completely liquid core. To prove this we loaded the capsules with CaCO _3_ or SiO _2_ particles. CaCO _3_ particles were nucleated directly in the preformed cores through the precipitation reaction of CaCl _2_ and Na _2_CO _3_, after step 2 of the protocol, by immersing the cores in a 0.33 M solution of CaCl _2_ and then adding under mixing a 0.33 M solution of Na _2_CO _3_, thus reaching supersaturation conditions and leading to the sudden precipitation of CaCO _3_ particles. Instead, SiO _2_ microparticles were loaded in the alginate/agarose solution before forming the cores at step 1 of the protocol.

In both cases, we dissolved the shell by placing the capsules into a 50 mM SCD solution to de-crosslink the shell. We then observed at the optical microscope the release of the loaded particles to determine whether the core was fully liquid or not (see Extended Data, video “ChargedCapsuleSiO2_core”) ^19^.

## 3 Results & discussion

### 3.1 Characterization of the capsules – shell thickness

The protocol described in section 2.2 led to the successful fabrication of the microcapsules. Figure 3 reports the microscopy images of two representative samples, acquired using transmitted illumination to emphasize the shell and measure its thickness.

**Figure 4.**
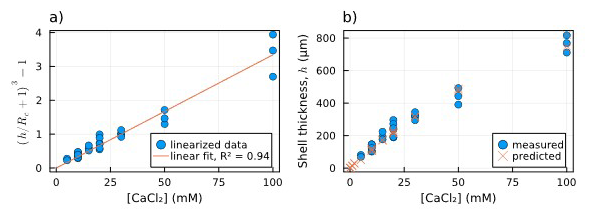
Dependence of shell thickness on [Ca ^2+^]: **a**) Linearized measured shell thickness (blue dots) alongside the linear fit of the data (orange line); **b**) Comparison between measured shell thickness (blue dots) and the thickness estimated using the parameter obtained from the fitting (orange crosses).

First, in Figure 4(a), the data related to the measured thicknesses of the hydrogel capsules are linearized through the left side of Equation 3 and plotted against CaCl _2_ concentration (which equals Ca ^2+^ concentration). Fitting the right side of Equation 3 to the linearized thickness data, we found a value of 0.033 mM ^−1^. In Figure 4(b) the comparison between the thickness values predicted by the fitted Equation 3 (orange crosses) and the measured ones (blue dots) is shown to be consistent. The fitted equation also provides a first estimation of the shell thickness for [CaCl _2_] < 5 mM, for which we could not measure the thickness directly.

**Figure 5.**
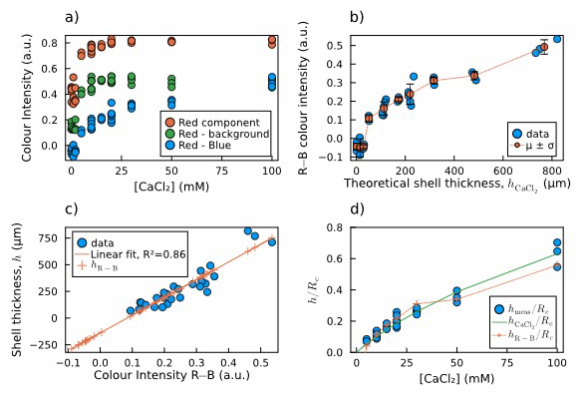
Estimation of shell thickness via colour analysis: **a**) Plot of the mean colour intensity in the selected area of each image: values of the red component (R, red dots), the red minus the background (R−bg, green dots), and the red minus the blue component (R−B, blue dots). **b**) Calculated R−B colour intensities and their mean and standard deviation for each concentration of calcium chloride *vs* the theoretical shell thickness calculated by Equation 2. **c**) Direct linear correlation between measured shell thickness and R−B colour intensity, including thickness predictions for both measured and non-measured sample. For the latter samples, the fitting predicts negative thickness values, which are not possible. **d**) Comparison of normalized shell thickness: measured (blue dots), predicted by Equation 2 (green solid line), and predicted by the R−B colour intensity (orange crosses and dotted line).

As explained in section 2.3, we extracted the average intensity of the red, green and blue components from an area corresponding to the centre of the capsules in the acquired images. Considering the reddish colouring given by iron oxide nanoparticles to the shell, we have chosen the red component, red minus background, and red minus blue colour intensities to be plotted against the concentration of CaCl _2_ soaked by the core of each particle ( Figure 5(a)).

Figure 5(a) shows that the values obtained as the difference between the red and the blue components (R−B) do not saturate at high [CaCl _2_], which instead happens in the other two cases. Therefore, we chose the R−B combination as the most reliable to be correlated to the shell thickness. In Figure 5(b), the measured R−B colour intensity is plotted against the theoretical shell thickness calculated by Equation 2. The plot is non-linear, with a threshold for low thickness values. The measured shell thickness values are plotted against the calculated R−B colour intensity, and the linear correlation between these two data was verified and calculated through a linear fit ( Figure 5(c), blue dots and orange line). The crosses lying on the left side of the fitting line correspond to the capsules for which the shell thickness was not measurable. The linear relation leads to the prediction of negative thickness values, which are obviously not possible, confirming our hypothesis of a threshold behaviour. This means that below a certain core [CaCl _2_] threshold, alginate shells did not form on top of the agarose cores.

Figure 5(d) shows the comparison between the two estimations and the measured thickness as normalized shell thickness vs concentration of CaCl _2_ soaked by the core. They are in good agreement, except for the points below 5 mM, for which the R−B colour intensity hints to the absence of a shell, which makes this second method more reliable for detecting the shell thickness rather than a simple linear extrapolation.

All images, data, and analysis scripts can be found in the Underlying Data ^20^.

### 3.2 Characterization of the capsules – core dissolution

The tests conducted with SiO _2_ and CaCO _3_ nanoparticles within the core of our capsules, aimed at evaluating the dissolution and diffusion of agarose, indicate that the core does not fully liquefy, likely because some agarose does not completely diffuse out. Specifically, when the capsules were placed back in the SCD solution after step 6, it was assumed that the agarose had melted due to the thermal bath. Under these conditions, we expected to observe the gradual release of SiO _2_ particles as the shell dissolved. Although this release does occur, a residue of gel, likely agarose, appears to persist (see Extended Data, video “ChargedCapsuleSiO2_core”) ^19^.

## 4 Conclusion

Most drug administration methods require high dosages and cause significant side effects, especially chemotherapy. Microrobots offer a potential solution by enabling precise navigation to hard-to-reach areas in the body. Ultra-deformable microrobots could help reach such areas and cross biological barriers. A promising design for ultra-deformable microrobots includes a thin, soft shell surrounding a liquid core. To fabricate our microrobots, we draw inspiration from leukocytes, which are highly deformable and able to move through small capillaries and cross the tiny junctions among the endothelial cells of the leaky vasculature in the tumour microenvironment. Here, we presented a method for fabricating capsules with a soft hydrogel shell and a liquid core as a preliminary step toward the realization of ultra-deformable microrobots. The method uses sacrificial agarose cores as a template to obtain alginate shells of different thicknesses, by soaking the cores in calcium chloride solutions of various concentrations, and liquid or insubstantial cores. Through the encapsulation of microparticles and the subsequent dissolution of the shell, we observed that the resulting core is not completely liquid, yet insubstantial enough not to considerably affect the deformability of the capsules.

We also developed a method to estimate the thickness of the capsules’ shell from microscopy images by measuring the colour intensity. This was especially meant to estimate the shell thickness of capsules with a shell that could not be directly recognized and measured. By these observations, we found that calcium chloride solutions of concentration ≤2 mM lead to capsule shells that are discontinuous or not forming at all, whereas we instead expected from Equation 2 to obtain thin shells.

Our fabrication method allows to make capsules with a shell thickness *h* down to ~0.1× *R _core_*. Therefore, by miniaturizing the sacrificial cores to the tens of microns range, we expect to obtain much thinner yet continuous shells (in the microns range). Indeed, the assessment of the fabrication method is performed on millimetre-sized capsules to ease the process and analysis, and further effort would be needed to adapt this method to make micron-sized capsules. Nevertheless, even if micrometric capsules are successfully produced, considering the realistically attainable shell thickness and contingent upon an assessment of their deformability, they might be able to move though channels (e.g. capillaries) smaller than their diameter, yet a concern remains as to whether these core-shell microcapsules could pass through junctions at the hundreds-of-nanometres scale. Indeed, while leukocytes and other cell types possess membranes with thicknesses on the order of nanometres, it appears that our current method cannot achieve nanometric shell thicknesses in tens-of-micron capsules. Although there are challenges to overcome, the development of such ultra-deformable microcapsules represents a promising approach to enhancing the efficacy and precision of drug delivery systems in biomedical applications. 

## Data Availability

Zenodo: Fabrication of Hydrogel Mini-Capsules as Carrier Systems - Underlying data. https://doi.org/10.5281/zenodo.8413186 ^20^. This project contains the following underlying data: images (folder containing the set of images used for the data analysis) data.csv (measurements acquired with the microscope software: Tag; CaCl2 concentration; shell thickness; core radius) Software_ThicknessAnalysis.jl (Julia script used for analysing the data) Functions_ThicknessAnalysis.jl (functions called in the script “Software_ThicknessAnalysis.jl”) readme.txt (file with the specifications on contents and used instrument/illumination conditions) Zenodo: Fabrication of Hydrogel Mini-Capsules as Carrier Systems - Extended data. https://doi.org/10.5281/zenodo.8413662 ^19^. This project contains the following extended data: ChargedCapsuleCaCO3.gif (time-lapse of an hydrogel capsule filled with CaCO3 to show the nature of the core through the release of the particles) ChargedCapsuleSiO2.jpg (image of an hydrogel capsule loaded with SiO2 microparticles) ChargedCapsuleSiO2_core.wmv (video of the particle in the picture - “ChargedCapsuleSiO2.jpg” after shell dissolution) TutorialBeadsFabrication.mov (video tutorial showing how the fabrication of the core-shell hydrogel capsules is realized) Data are available under the terms of the Creative Commons Attribution 4.0 International license (CC-BY 4.0).
