# Fabrication of hydrogel mini-capsules as carrier systems

**DOI:** 10.12688/openreseurope.16723.3

**Published:** 2025-07-25

**Authors:** Elisa Roberti, Gaia Petrucci, Francesco Bianciardi, Stefano Palagi

**Affiliations:** 1 The BioRobotics Institute, Sant'Anna School of Advanced Studies, Pisa, Tuscany, 56025, Italy; 2 Department of Excellence in Robotics & AI, Sant'Anna School of Advanced Studies, Pisa, Tuscany, 56127, Italy

**Keywords:** alginate, agarose, core-shell, hydrogel capsules, microrobotics

## Abstract

Conventional drug administration often results in systemic action, thus needing high dosages and leading to potentially pronounced side effects. Targeted delivery, employing carriers like nanoparticles, aims to release drugs at a target site, but only a small fraction of nanoparticles reaches it. Microrobots could overcome this issue, being guided to hard-to-reach sites and locally delivering payloads. To enhance their functionality, we propose microrobots made as deformable capsules with hydrogel shells and aqueous cores, having the potential added advantages of biocompatibility, permeability, and stimulus-responsiveness. Endowing microrobots with deformability could allow them to navigate inside capillaries and cross barriers to finally reach the target site. In this study, we present a cost-effective method for fabricating core-shell structures without the use of organic solvents, surfactants, or extreme pH conditions. First, a mixture of hydrogels, agarose and alginate, is dripped into a calcium chloride solution to form beads. After they are loaded with calcium ions at different concentrations, they are immersed in an alginate solution to form the shell. Finally, the beads are heated to let the agarose melt and diffuse out, leaving a liquid core. By varying the concentration of calcium ions, we obtain shells of different thicknesses. We have correlated the measured shell thickness to its colour intensity and extrapolated to estimate the thickness of shells too thin to be measured directly. This allowed us to conclude that no continuous shells forms below a certain calcium chloride concentration. For higher concentrations, although the core may remain partially gelled, continuous shells successfully form. To qualitative assess core-shell capsule deformability, we forced them through a tube with an inner diameter ~1.6 times smaller than the average capsule diameter. The capsules deformed to pass through the constriction while maintaining structural integrity. Therefore, our fabrication method offers a promising platform for applications in drug delivery, encapsulation systems, and microrobotics.

## Plain language summary

Conventional drug administration methods often lead to widespread effects in the body and dosage-related side effects. Targeted drug delivery aims to release drugs precisely where they’re needed by using carriers. However, only a few of them actually reach the intended target. To address this issue, scientists are exploring microrobots, tiny machines that can reach places deep inside the body. We design microrobots as soft liquid-filled capsules. In our research, we’ve developed an affordable method to make these capsules without using harsh chemicals. The obtained capsules have a controllable shell thickness and a liquid or almost liquid core. This makes our capsules promising candidates as microrobot bodies or as drug delivery carriers.

## 1 Introduction

Most routes of drug administration ( *e.g.* intravenous) lead to systematic action of the drug, as local administration is possible only for suitable diseases. In many cases, this requires much higher dosages than those needed if the administration could be local, resulting in much more significant side effects ^1^. This is particularly problematic for chemotherapy. To address this issue, conventional drug delivery approaches aim to improve aqueous solubility and chemical stability of the drug, increase its pharmacological activity and reduce side effects while maintaining the therapeutic concentrations at the target site ^2^. A suitable approach to reduce drugs’ side effects consists in adopting carriers, such as nanoparticles, that could release the drug only at the target site or to target tissues or cells, which is known as targeted drug delivery ^3, 4^. The drug loading in nanosystems could be categorized into three main strategies: pre-loading, co-loading, and post-loading. Pre-loading involves forming drug nanoparticles first and then coating them with a stabilizing shell. Co-loading, the most common method, incorporates the drug during the nanocarrier’s while post-loading, introduces the drug into already formed nanocarriers ^5^. Whereas nanoparticles-based targeted drug delivery has proved to enhance the therapeutic index and reduce adverse side effects, there are still open issues related to drugs toxicity, selectivity and dosage. One key problem is that only a tiny fraction of the drug-loaded nanoparticles reaches the target site (e.g. a solid tumour) ^6^.

Microrobots have been proposed to overcome these issues ^7^. The main feature of these tiny robotic devices is their controlled navigation, which could allow them to be guided to hard-to-reach target sites inside the human body. Microrobotics could thus become a non-invasive approach to locally administer drugs (or drug-loaded carriers) at sites that are not currently suited to local administration. Although recent advances in microrobotics for drug delivery applications, tracking and guiding microrobots *in vivo* remains challenging, and their ability to move through body tissues and cross biological barriers is still limited ^7^. To make microrobots ultra-deformable and able of moving across tissues and barriers, a possible design consists of a thin, soft shell enveloping a liquid core ^8, 9^. Ultra-deformability refers to the ability of these microrobots to deform substantially and reversibly allowing them to pass through openings ^10– 13^. We take inspiration from leukocytes, particularly neutrophils and macrophages, which are sufficiently deformable to move inside small capillaries (< 5 µm) that reach solid tumours, travel through the interstitial spaces of tissues (< 1 µm), and extravasate through hundreds-nanometres junctions between cells of the leaky vasculature in the tumour microenvironment ^6^. Moreover, the microrobots’ building materials should be biocompatible or biodegradable. Additionally, the shell should be permeable and possibly stimuli-responsive, releasing.

Core-shell and hollow particles can be prepared using a variety of methods ^14, 15^, including: Layer-by-Layer (LbL) ^16, 17^, sol-gel ^18, 19^, solvent evaporation, spray-drying ^20^, double emulsions (O/W/O or W/O/W) ^21^. However, none of these methods is suited to the fabrication of thin-shell gel-based microrobots we have conceived, which requires mild process conditions to avoid damages to the potential payloads.

In this article, we introduce a method for fabricating core-shell capsules that have a liquid aqueous core and a hydrogel shell, which involves no organic solvents, surfactants, or low/high pHs. We exploit the different gelation mechanisms of alginate and agarose to create the core-shell capsules. Specifically, alginate crosslinks in the presence of calcium ions, while agarose gelate at room temperature. We report the characterization of the obtained core-shell capsules, with a focus on the shell thickness and its dependence on the process parameters. We also qualitatively evaluated the deformability of the core-shell capsules by forcing the capsules through a silicone tube. The proposed process holds potential also for the fabrication of micro-capsules and core-shell particles for different applications, including drug-delivery systems other than microrobotics ones.

## 2 Methods

### 2.1 Materials

Sodium alginate (CAS 9005-38-3), agarose (CAS 9012-36-6), sodium citrate dihydrate (SCD)(CAS 6132-04-3), calcium chloride (CAS 10043-52-4) and iron (III) oxide nanopowder <50 nm (CAS 1309-37-1) were purchased from Merck. 0.45 μm filters (CAS 514-1267) were purchased from VWR ^®^. silicone tube (CAS 667-8441) was purchased from RS PRO. 10 mL Syringes (CAS MDSS10SE) were purchased from Terumo ^®^. MilliQ water is used unless differently stated (Elix ^®^ Advantage 10 Water Purification System).

To fabricate the shell we have chosen alginate, a natural polymer which shows attractive properties such as biocompatibility, low cost, ease of gelation, inert nature ^22^. Alginate undergoes a reversible gelation process when divalent ions ( *e.g.* Ca ^2+^, Ba ^2+^ or Sr ^2+^) are added to the polymeric solution and it returns to the original liquid form when exposed to chelating agents that bind the divalent ions with a higher affinity constant ^23^.

### 2.2 Preparation of the core-shell particles

**Figure 1.**
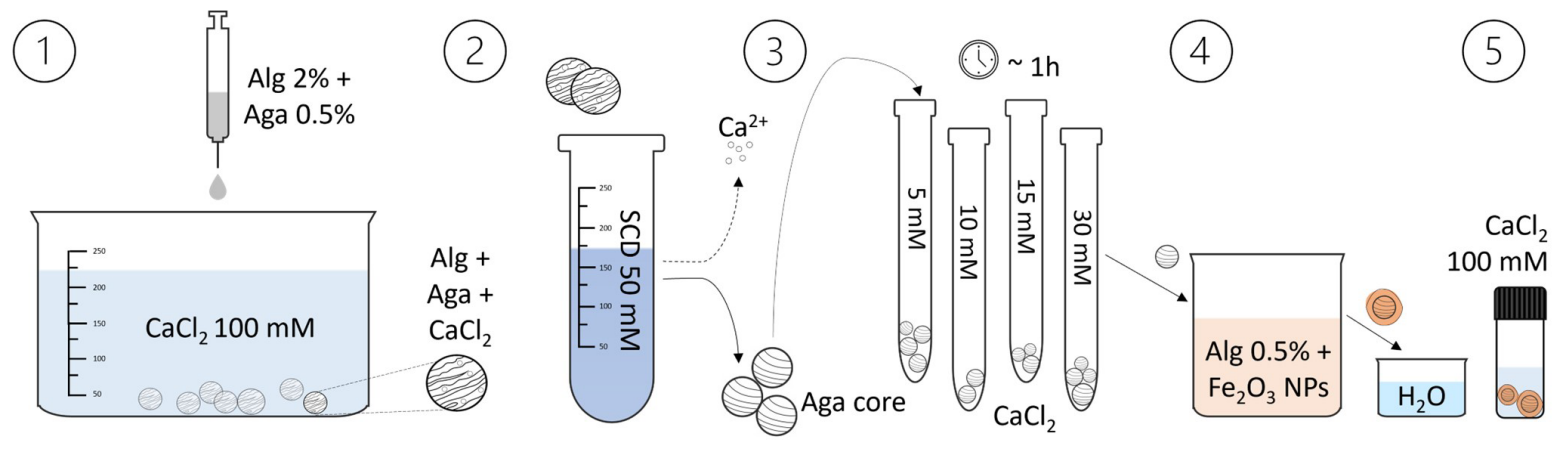
Illustration of the core-shell particles fabrication process. The core-shell beads preparation involves the dripping of an agarose-alginate mixture in a CaCl _2_ solution, followed by washing and transferring the beads to a sodium citrate dihydrate (SCD) solution. After loading them with Ca ^2+^ ions (by equilibration in CaCl _2_ solutions of different concentrations), the beads are placed in an alginate solution containing Fe _2_O _3_ nanoparticles to form the shell. Subsequently, they are washed and transferred to another CaCl _2_ solution to consolidate the shell. By heating the beads, the agarose liquefies and diffuses out, resulting in a liquid core within the shell.

The process for preparing the core-shell beads is depicted in Figure 1 and a tutorial video can be found in the Extended Data ^24^.

1. The agarose-alginate water solution is prepared mixing a 1% w/v agarose solution (previously heated at 95°C to promote dissolution) and a 4% w/v alginate solution to have overall concentrations of 0.5% agarose and 2% alginate. The obtained warm solution is filtered (0.45 μm filters) and then it is added drop-wise to a CaCl _2_ 100 mM solution using a 10 mL syringe (Terumo ^®^) to form gel beads because of the immediate crosslinking of alginate by calcium ions. The beads are kept in the CaCl _2_ solution for about 10 minutes to complete the crosslinking.
2. The beads are then washed with 100 ml of water to remove the excess of CaCl _2_, transferred into a 50 mM solution of sodium citrate dihydrate (SCD) and kept overnight. SCD is meant to chelate Ca ^2+^ ions and thus de-crosslink the alginate template, leaving beads of just agarose.
3. The agarose beads are then washed again with water and transferred in CaCl _2_ solutions at different concentrations (ranging from 0.1 mM to 100 mM).
4. After 1 hour of equilibration in the CaCl _2_ solution, the beads are picked up and briefly placed on a filter paper to remove the excess solution from the surface. They are then immersed for 15 minutes in a solution of alginate 0.5% w/v to form an alginate shell around the agarose bead templates. The alginate solution contains 0.2 mg/ml of 50 nm Fe _2_O _3_ nanoparticles to enhance the shell visualization under the optical microscope.
5. After 15 minutes, the core-shell structures are briefly rinsed in water and then transferred in a 100 mM CaCl _2_ solution to complete the crosslinking of the alginate shell.
6. To obtain a liquid core, the core-shell structures are placed in a thermal bath at a temperature of 90/95°C for 2–7 hours, allowing the agarose to liquefy and diffuse out, leading to microcapsules with an alginate shell and a liquid aqueous core.

We immersed capsules purposely made using cores loaded with particles (CaCO _3_ or SiO _2_) in a SCD solution to de-crosslink the shell and assess the core liquidity. The release of the particles from the capsules highlighted that the cores were insubstantial after 6–7 hours of thermal bath, although we still observed a light gel residue (see Extended Data, video “ChargedCapsuleSiO2_core”) ^24^.

### 2.3 Capsules characterization

***2.3.1 Shell formation and thickness***

We characterized the beads using an Hirox optical digital microscope (Hirox HRX-01) and analysing the images by two different approaches: i) direct measure of the shell thickness – this method is however challenging for thin shells; ii) indirect measure based on shell colour.

**Figure 2.**
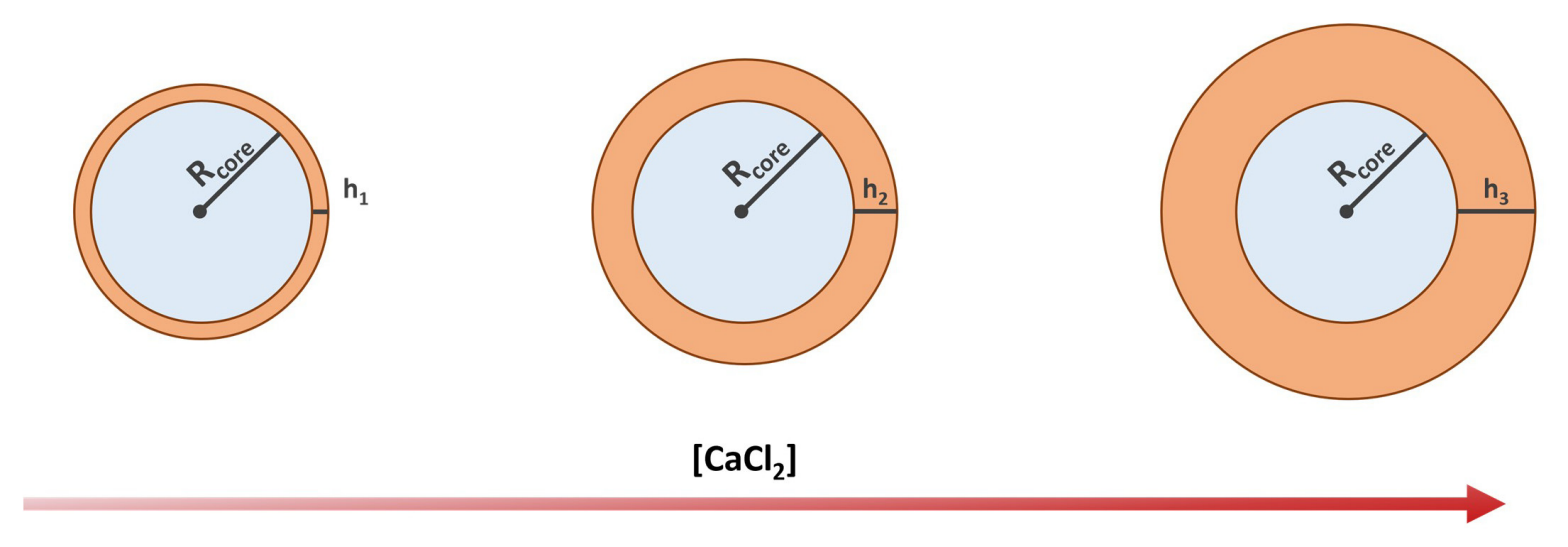
Schematics showing the expected shell thickness increase with the increasing concentration of calcium chloride.

We assume the volume *V _s_*, and thus the thickness of the alginate shell, to be proportional to the amount of Ca ^2+^ ions loaded into the core (see Figure 2), such that

$$\;V_{s}=\alpha \;n_{{\text{Ca}}^{\text{2+}}}\;(1)$$

where *α* is a proportionality constant (to be determined) and $n_{{\text{Ca}}^{\text{2+}}}=V_{c}[{\text{Ca}}^{2+}]$ is the number of Ca ^2+^ ions moles in the core ([Ca ^2+^] is assumed to be equal to the concentration of the CaCl _2_ solution the cores have been soaked in before forming the alginate shell). Considering $V_{c}=\frac{4}{3}\pi R_{c}^{3}$ (where *R _c_* is the core radius) and $V_{s}=\frac{4}{3}\pi [{\left(R_{c}+h\right)}^{3}-R_{c}^{3}]$ (where is *h* the thickness of the shell), we obtain

$$h=R_{c}\left(\sqrt[{3}]{\alpha [{\text{Ca}}^{\text{2+}}]+1}-1\right)\;(2)$$

**Figure 3.**
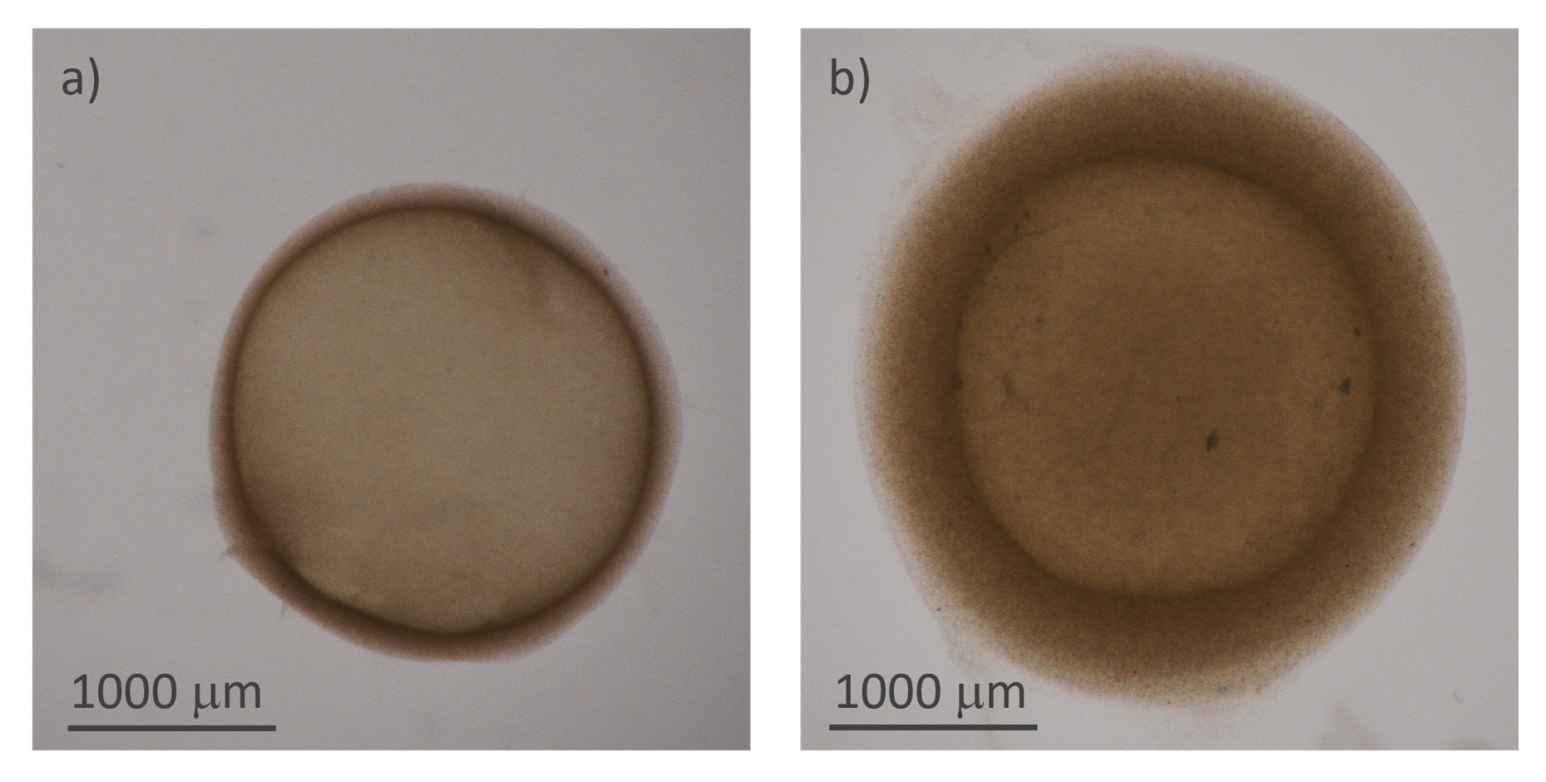
Structure of the hydrogel microcapsules: **a**) and **b**) optical microscope images of two capsules of different thicknesses.

When the core is immersed in a solution at a fixed alginate concentration, the release of Ca ^2+^ ions induces the crosslinking of alginate around the cores. This produces a shell whose thickness depends on the number of Ca ^2+^ ions previously absorbed by the core. Therefore, we assume n Ca ^2+^ is and thus to *V _s_*. We then fabricated batches of capsules by soaking the agarose cores in CaCl _2_ solutions at concentrations ranging between 0.1 and 100 mM (0.1, 1, 2, 5, 10, 15, 20, 30, 50 and 100 mM, three to five samples for each concentration) – see Figure 3. We stained the shell by adding iron oxide nanoparticles to the alginate solution to enhance its detection and ease the measurement of its thickness (particles were chosen instead of a dye, as most dyes would diffuse away and/or stain the core too).

We first measured the shell thicknesses using the measuring tool of the digital optical microscope’s software. To perform a linear fit and obtain an estimate for *α*, we rearranged Equation 2 to obtain an equation of the form *y* = *ax*, leading to:


$${\left(\frac{h}{R_{c}}+1\right)}^{3}-1=\alpha [{\text{Ca}}^{\text{2+}}]\;(3)$$

Once obtained a value for *α*, Equation 2 can be used to calculate the expected shell thickness as a function of the CaCl _2_ solution concentration. Therefore, we estimated the thickness of the shells too thin to be directly measured by extrapolating values from the fitted curve.

To allow for estimating the thickness of such thin shells from actual images of the beads, we correlated the shell thickness to its colour intensity (given by the iron oxide nanoparticles).To have consistent measurements of the colour intensity, we set and kept fixed the parameters for the microscope images acquisition (illumination, camera settings). The parameters values were chosen to obtain good quality images with all the inspected thicknesses. The image processing develops as follow:

1. each image of the set is segmented creating two clusters: one with all the pixels corresponding to the capsules, and a second one with the background;
2. in each image, a small portion at the centre of the capsule cluster is automatically selected, and the RGB values of the corresponding pixels are extracted;
3. in each image, an average value for each of the three colour channels is calculated over the selected region;
4. in each image, average channel values corresponding to the background are also calculated;
5. from the colour intensities extracted at point 3 and 4, the red channel value, the value of the red channel minus the background, and the value of the red channel minus the blue channel are calculated and related to the CaCl _2_ concentration and to the theoretical shell thickness (calculated with Equation 2);
6. a linear fit of the measured shell thickness over the measured R−B colour intensity is performed, obtaining a relation to estimate the shell thickness from the colour intensity.

From this we again extrapolate the expected thickness for the non-measurable shells and compare the predictions with those obtained from Equation 2.

***2.3.2 Core dissolution***

To ensure good degree of deformability, our core-shell capsules should consist of a highly soft shell and a predominantly liquid core, as previously mentioned. Step 2 and step 6 of the fabrication protocol are indeed aimed at removing the alginate and the agarose, respectively, from the core. Step 6, in particular, aims at melting the agarose and letting it diffuse out through the alginate shell, ideally leading to a completely liquid core. To assess the consistency of the core, we loaded the capsules with CaCO _3_ or SiO _2_ particles and observed their release from the core. CaCO _3_ particles were nucleated directly in the preformed cores through the precipitation reaction of CaCl _2_ and Na _2_CO _3_, after step 2 of the protocol, by immersing the cores in a 0.33 M solution of CaCl _2_ and then adding under mixing a 0.33 M solution of Na _2_CO _3_, thus reaching supersaturation conditions and leading to the sudden precipitation of CaCO _3_ particles. Instead, SiO _2_ microparticles were loaded in the alginate/agarose solution before forming the cores at step 1 of the protocol.

In both cases, we dissolved the shell by placing the capsules into a 50 mM SCD solution to de-crosslink the shell. We then observed at the optical microscope the release of the loaded particles to determine whether the core was fully liquid or not (see Extended Data, video “ChargedCapsuleSiO2_core”) ^24^.

***2.3.3 Deformability tests***

**Figure 4.**
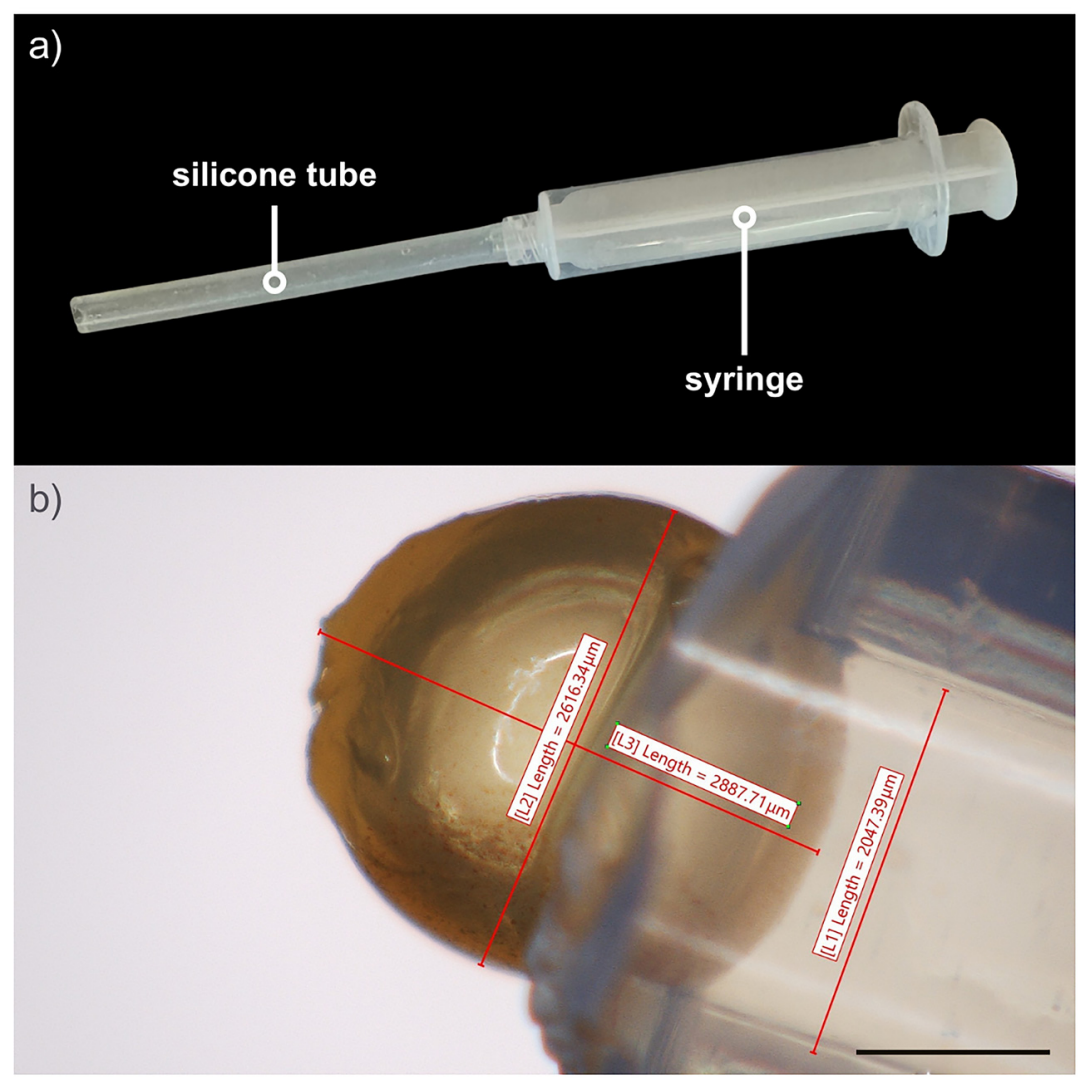
Qualitative deformability tests. **a**) experimental setup used to assess the deformability of core-shell capsules. It consists of a silicone tube with an internal diameter of 1.6 mm connected to a plastic syringe. The capsule is drawn from the open end of the tube opposite to the syringe; **b)** the internal diameter of the tube is smaller than the capsule size. Scale bar: 1000 μm.

To qualitatively evaluate the deformability of our core–shell capsules, we conducted tests on two types of capsules: capsules with ideally liquid cores, obtained through thermal treatment at 95 °C, and core-shell particles with solid cores, produced by omitting the final heating step (see step 6 in Section 2.2). The test consists of forcing these capsules through a silicone tube with an internal diameter of 1.6 mm using a syringe (see Figure 4). Specifically, we tested a few representative capsules per type with a shell composed of alginate crosslinked with 30 mM and 50 mM CaCl₂ and with a diameter larger than the inner diameter of the silicone tube. The capsules were drawn into the tube using a 10 mL syringe, and their passage through the tubing was observed using a digital optical microscope (Hirox HRX-01).

## 3 Results & discussion

### 3.1 Characterization of the capsules – shell thickness

The protocol described in Section 2.2 led to the successful fabrication of the microcapsules. Figure 3 reports the microscopy images of two representative samples, acquired using transmitted illumination to emphasize the shell and measure its thickness.

**Figure 5.**
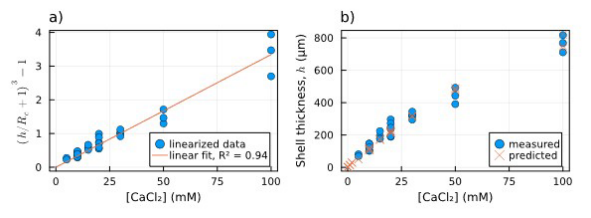
Dependence of shell thickness on [Ca ^2+^]: **a**) Linearized measured shell thickness (blue dots) alongside the linear fit of the data (orange line); **b**) Comparison between measured shell thickness (blue dots) and the thickness estimated using the parameter obtained from the fitting (orange crosses).

First, in Figure 5(a), the data related to the measured thicknesses of the hydrogel capsules are linearized through the left side of Equation 3 and plotted against CaCl _2_ concentration (which equals Ca ^2+^ concentration). Fitting the right side of Equation 3 to the linearized thickness data, we found a value of 0.033 mM ^−1^. In Figure 5(b) the comparison between the thickness values predicted by the fitted Equation 3 (orange crosses) and the measured ones (blue dots) is shown to be consistent. The fitted equation also provides a first estimation of the shell thickness for [CaCl _2_] < 5 mM, for which we could not measure the thickness directly.

**Figure 6.**
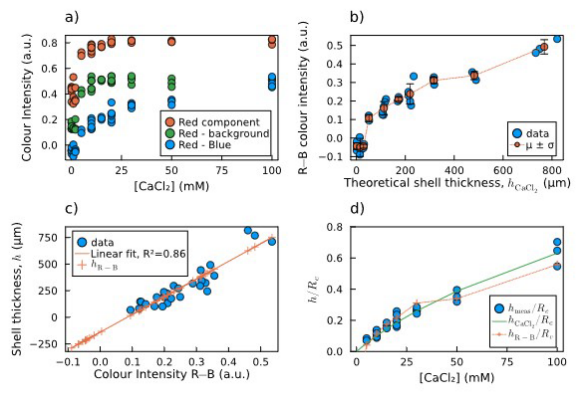
Estimation of shell thickness via colour analysis: **a**) Plot of the mean colour intensity in the selected area of each image: values of the red component (R, red dots), the red minus the background (R−bg, green dots), and the red minus the blue component (R−B, blue dots). **b**) Calculated R−B colour intensities and their mean and standard deviation for each concentration of calcium chloride *vs* the theoretical shell thickness calculated by Equation 2. **c**) Direct linear correlation between measured shell thickness and R−B colour intensity, including thickness predictions for both measured and non-measured sample. For the latter samples, the fitting predicts negative thickness values, which are not possible. **d**) Comparison of normalized shell thickness: measured (blue dots), predicted by Equation 2 (green solid line), and predicted by the R−B colour intensity (orange crosses and dotted line).

As explained in Section 2.3, we extracted the average intensity of the red, green and blue components from an area corresponding to the centre of the capsules in the acquired images. Considering the reddish colouring given by iron oxide nanoparticles to the shell, we have chosen the red component, red minus background, and red minus blue colour intensities to be plotted against the concentration of CaCl _2_ soaked by the core of each particle ( Figure 6(a)).

Figure 6(a) shows that the values obtained as the difference between the red and the blue components (R−B) do not saturate at high [CaCl _2_], which instead happens in the other two cases. Therefore, we chose the R−B combination as the most reliable to be correlated to the shell thickness. In Figure 6(b), the measured R−B colour intensity is plotted against the theoretical shell thickness calculated by Equation 2. The plot is non-linear, with a threshold for low thickness values. The measured shell thickness values are plotted against the calculated R−B colour intensity, and the linear correlation between these two data was verified and calculated through a linear fit ( Figure 6(c), blue dots and orange line). The crosses lying on the left side of the fitting line correspond to the capsules for which the shell thickness was not measurable. The linear relation leads to the prediction of negative thickness values, which are obviously not possible, confirming our hypothesis of a threshold behaviour. This means that below a certain core [CaCl _2_] threshold, alginate shells did not form on top of the agarose cores.

Figure 6(d) shows the comparison between the two estimations and the measured thickness as normalized shell thickness vs concentration of CaCl _2_ soaked by the core. They are in good agreement, except for the points below 5 mM, for which the R−B colour intensity hints to the absence of a shell, which makes this second method more reliable for detecting the shell thickness rather than a simple linear extrapolation.

All images, data, and analysis scripts can be found in the Underlying Data ^25^.

### 3.2 Characterization of the capsules – core dissolution

The tests conducted with SiO _2_ and CaCO _3_ nanoparticles within the core of our capsules, were designed solely to evaluate the dissolution of the core.. Theyindicate that the core does not fully liquefy, likely because some agarose does not completely diffuse out. Specifically, when the capsules were placed back in the SCD solution after step 6, it was assumed that the agarose had melted due to the thermal bath. Under these conditions, we expected to observe the gradual release of SiO _2_ particles as the shell dissolved. Although this release does occur, a residue of gel, likely agarose, appears to persist (see Extended Data, video “ChargedCapsuleSiO2_core”) ^24^.

### 3.3 Deformability test

**Figure 7.**
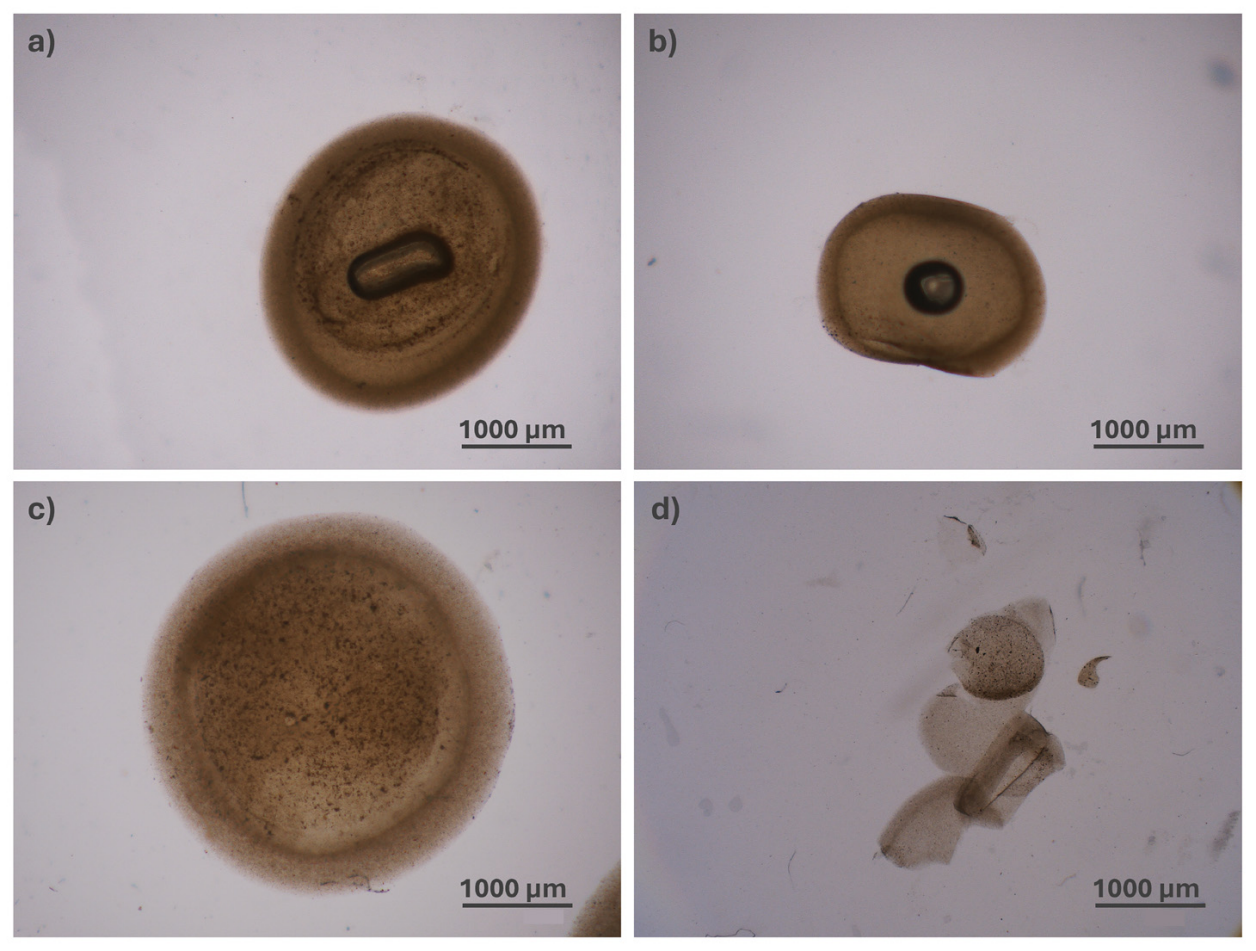
Capsules before 1 **a**), **c**) and after **b**), **d**) the passage through the silicone tube. The core-shell capsule with a semi-liquid core composed of a shell made of alginate crosslinked with 50 mM CaCl₂, shown before **a**) and after **b**) passage through the silicone tube. The capsule clearly deforms to pass through the silicone tube while maintaining the integrity of their structure. In contrast, the core-shell particle with a solid core completely lost its structural integrity after passing through the silicone tube ( Figure 7d).

Deformability tests revealed that the core-shell capsules fabricated using the proposed protocol are highly deformable, even though the core is not fully liquid. We evaluated two capsules with a shell composed of alginate crosslinked with 50 mM CaCl _2_ and two with a shell crosslinked with 30 mM CaCl _2_. For comparison, we also tested the deformability of four core-shell particles with a solid core – i.e., not subjected to step 6 of the protocol – while maintaining the same shell composition: two with a shell crosslinked with 30 mM and two with 50 mM CaCl _2_). Following passage through the constriction, three out of the four capsules subjected to step 6 (core dissolution) retained their structural integrity under both shell conditions (see Figure 7b), as reported in Table 1. However, in one of the two capsules with a shell crosslinked at 30 mM, capsule rupture occurred during syringe drawn. In contrast, all solid-core particles were larger than semi-liquid core ones and failed to maintain their structural integrity.

**Table 1.** Dimensions of core-shell capsules and particles, and outcome of the deformability test. The table reports the diameters of the tested core-shell capsules and the corresponding outcome after passing through the silicone tubing. In case #1, the capsule broke during the drawing phase, prior to undergoing the actual deformability test.

	Core-shell capsules (semi-liquid core)			
	30mM		50mM	
	#1	#2	#1	#2
Diameter (μm)	1295	1297	2043	2056
Survived	no	yes	yes	yes

## 4 Conclusion

Most drug administration methods require high dosages and cause significant side effects, especially chemotherapy. A promising solution lies in drug-delivery systems (e.g. nanosystems) where the drug is encapsulated to reduce both dosage and toxicity, while also enabling targeted release. Among these systems, nanoparticles are widely used. However a major limitation is that, because administration is usually systemic, only a small fraction (<1%) of the administered dose typically reaches the target site. Microrobots may offer a solution by enabling precise navigation to hard-to-reach areas in the body and, thus, local administration of drug-loaded nanoparticles. Making microrobots ultra-deformable microrobots could help reach such areas and cross biological barriers. A promising design for such microrobots includes a thin, soft shell surrounding a liquid core. In this work, we presented a method for fabricating capsules with a soft hydrogel shell and a liquid aqueous core as a preliminary step toward the realization of ultra-deformable microrobots. The method uses sacrificial agarose cores as a template to obtain alginate shells of different thicknesses and liquid or insubstantial cores. Through the encapsulation of microparticles and the subsequent dissolution of the shell, we observed that the resulting core is not completely liquid, yet insubstantial enough not to considerably affect the deformability of the capsules. This was further demonstrated by passing one representative capsule through a silicone tube with a diameter smaller than that of the capsule. Following passage, the capsule maintained its structural integrity demonstrating its ability to undergo significant deformation.

We also correlated the thickness of our capsules’ shell from microscopy images to their colour intensity. This was especially meant to estimate the shell thickness of capsules with a shell that could not be directly recognized and measured from microscope images. By these observations, we concluded that calcium chloride solutions of concentration ≤2 mM lead to capsule shells that are discontinuous or not forming at all instead of the thin shells we expected from Equation 2. While we cannot definitively rule out the presence of an alginate shell, we strongly suspect that its formation is minimal or incomplete and, in either case, inadequate for the intended purpose of this study.

Our fabrication method allows to make capsules with a shell thickness *h* down to ~0.1× *R _core_.* Therefore, by miniaturizing the sacrificial cores to the tens of microns range, we expect to obtain much thinner yet continuous shells (in the microns range). Indeed, the assessment of the fabrication method is performed on millimetre-sized capsules to ease the process and analysis, and further effort would be needed to adapt this method to make micron-sized capsules. The miniaturization of the capsules is primarily limited by the use of syringe needles for the initial formation of the sacrificial cores. For this reason, alternative methods that enable the production of smaller cores (e.g. microfluidics or spray techniques) should be investigated. Nevertheless, even if micrometric capsules are successfully produced, considering the realistically attainable shell thickness and contingent upon an assessment of their deformability, they might be able to move through channels (e.g. capillaries) smaller than their diameter, yet a concern remains as to whether these core-shell microcapsules could pass through junctions at the hundreds-of-nanometres scale. Indeed, while leukocytes and other cell types possess membranes with thicknesses on the order of nanometres, it appears that our current method cannot achieve nanometric shell thicknesses in tens-of-micron capsules.

In view of future applications, depending on the specific drug to be delivered, adaptations to the fabrication protocol may be required, for example, the use of low-melting-point agarose to avoid exposure to elevated temperatures that could compromise the stability of thermosensitive cargoes. We envision ultra-deformable microrobots capable of moving through body tissues and locally administering smart nanomedicines. Such administration could be triggered by external inputs or specific local conditions, e.g. altered pH. Although there are challenges to overcome, the development of ultra-deformable microrobots represents a promising approach to enhancing the efficacy and precision of drug delivery systems in biomedical applications.

## Data Availability

Zenodo: Fabrication of Hydrogel Mini-Capsules as Carrier Systems - Underlying data. https://doi.org/10.5281/zenodo.8200188 ^25^. This project contains the following underlying data: images (folder containing the set of images used for the data analysis) microscopy_measurments.csv (measurements acquired with the microscope software) Software_ThicknessAnalysis.jl (main script for reproducing the data analysis) Functions_ThicknessAnalysis.jl (collection of functions for the data analysis) Manifest.toml and Project.toml (computational environment files) _init_.jl (utility script for reproducing the computational environment) README.md Zenodo: Fabrication of Hydrogel Mini-Capsules as Carrier Systems - Extended data. https://doi.org/10.5281/zenodo.8413661 ^24^. This project contains the following extended data: ChargedCapsuleCaCO3.gif (time-lapse of an hydrogel capsule filled with CaCO3 to show the nature of the core through the release of the particles) ChargedCapsuleSiO2.jpg (image of an hydrogel capsule loaded with SiO2 microparticles) ChargedCapsuleSiO2_core.wmv (video of the particle in the picture - “ChargedCapsuleSiO2.jpg” after shell dissolution) TutorialBeadsFabrication.mov (video tutorial showing how the fabrication of the core-shell hydrogel capsules is realized) TimelapseCapsuleDyeReleased.png (release of a green dye from one capsule monitored over 30 minutes) CapsuleInsideSiliconeTube.mov (video of the capsule passing through the silicone tube) LowMeltAgaroseCapsule.png (images of a core-shell capsule fabricated using low-melting agarose) Data are available under the terms of the Creative Commons Attribution 4.0 International license (CC-BY 4.0).
