# Supplementary material for: Fabrication of hydrogel mini-capsules as carrier systems
Source: Open Res Eur. 2025 Jul 25;3:191. Originally published 2023 Nov 7. [Version 3] doi: 10.12688/openreseurope.16723.3 (PMC11605173; doi:10.12688/openreseurope.16723.3)
Supplement: Peer review history

# Reviewer report: Reviewer response for version 3

**DOI:** 10.21956/openreseurope.22698.r57059

**Published:** 2025-08-06

**Authors:** Min-Soo Kim

**Affiliations:** 1 ETH Zurich, Zurich, Switzerland

The authors have addressed my concerns.

Is the study design appropriate and does the work have academic merit?

Yes

Is the work clearly and accurately presented and does it cite the current literature?

Yes

If applicable, is the statistical analysis and its interpretation appropriate?

Partly

Are all the source data underlying the results available to ensure full reproducibility?

Partly

Are the conclusions drawn adequately supported by the results?

Partly

Are sufficient details of methods and analysis provided to allow replication by others?

Partly

Reviewer Expertise:

NA

I confirm that I have read this submission and believe that I have an appropriate level of expertise to confirm that it is of an acceptable scientific standard.

# Reviewer report: Reviewer response for version 2

**DOI:** 10.21956/openreseurope.20144.r48108

**Published:** 2025-01-10

**Authors:** Min-Soo Kim

**Affiliations:** 1 ETH Zurich, Zurich, Switzerland

This manuscript introduces a fabrication method for hydrogel-based core-shell microcapsules intended for drug delivery applications. A parametric study on shell thickness, influenced by the crosslinking agent (CaCl _2_) concentration, revealed that the shell thickness varies between 100 µm and 800 µm with ~2 mm of core diameter. The proposed manufacturing technique offers an advantage as it does not require organic solvents, surfactants, or extreme pH conditions. The vision-based shell thickness measurement strategy also presents potential utility for microcapsule research. Below are concerns the authors should address:

 1.  Additional mechanical tests are necessary to demonstrate the deformability of the microcapsules, such as measuring deformation under applied pressure or force.

 2. The lack of shell formation at lower CaCl _2_ concentrations (threshold behavior) should be supported by additional experimental evidence to substantiate this claim.

 3. In practical applications, how much drug can be loaded into the microcapsules as a percentage of their volume? What types of drugs can be incorporated into the core? How is the alginate shell dissolved at the targeted site, and what alternatives to sodium citrate dihydrate can be used for shell dissolution?

 4. The authors should clarify the image-based thickness measurement method. It is essential to clarify whether the red-blue channel intensity is measured solely at the center point or across a region between the center and the background.

 5. The fabrication steps for loading core materials (e.g., SiO _2_ or CaCO _3_) described in Section 2.3.2 should be moved to precede Section 2.3 for better logical flow.

Is the study design appropriate and does the work have academic merit?

Yes

Is the work clearly and accurately presented and does it cite the current literature?

Yes

If applicable, is the statistical analysis and its interpretation appropriate?

Partly

Are all the source data underlying the results available to ensure full reproducibility?

Partly

Are the conclusions drawn adequately supported by the results?

Partly

Are sufficient details of methods and analysis provided to allow replication by others?

Partly

Reviewer Expertise:

Microrobotics, Microfabrication, Micro mechancial test

I confirm that I have read this submission and believe that I have an appropriate level of expertise to confirm that it is of an acceptable scientific standard, however I have significant reservations, as outlined above.

# Reviewer report: Reviewer response for version 2

**DOI:** 10.21956/openreseurope.20144.r46291

**Published:** 2024-12-05

**Authors:** Bradley Nelson, Fabian Landers

**Affiliations:** 1 Multi-Scale Robotics Lab, Zurich, Switzerland; 2 D-MAVT, ETH Zurich, Zürich, Zurich, Switzerland

The manuscript introduces a fabrication method for hydrogel-based core-shell capsules designed for drug delivery. The authors present an intriguing approach to achieving a liquid core without relying on extreme pH conditions or organic solvents, which is advantageous for sensitive drugs. Using different cross-linking mechanisms is an elegant solution for creating core-shell structures. Additionally, introducing a shell thickness measurement protocol is a valuable contribution that could benefit other researchers in the field.

 The authors should address the following comments:

1. The authors highlight the benefits of "topical" drug administration at several points. Is the topical route going to be the primary route of delivery? If not, the term "local" may be more suited in this context.
2. In the first part of the introduction, the authors describe the advantages of local drug delivery. While these statements are valid, adding more references would better illustrate the scope and potential of solving this challenge.
3. The authors state that a double-emulsion process is unsuitable for fabricating ultra-deformable microrobots. Clarification is needed here: why exactly is this process not suitable?
4. It is unclear which specific drug the authors envision integrating into their microrobots. While the fabrication process avoids extreme pH conditions and organic solvents, it requires high temperatures (90–95 °C for 2–7 hours) to melt the core. What types of drugs would be compatible with these conditions? Small-molecule drugs could be a good candidate, but would diffusion out of the microrobot pose a problem? The authors mention avoiding dyes for shell staining due to diffusion challenges, which raises concerns about potential drug leakage.
5. The proposed protocol for measuring shell thickness is an interesting and innovative method that addresses a critical issue. However, its validity should be confirmed using an established method for comparison.
6. The dissolution of the microrobots is achieved by immersing them in the SCD solution. How could such conditions be replicated in vivo?
7. An analysis of the polydispersity of the microrobots should be added to provide insight into the fabrication process.
8. Mechanical testing of the microrobots' deformability is necessary to substantiate the claim of ultra-deformability. Simple tests like flushing the microrobots through various pore sizes would provide supporting evidence.
9. Finally, the potential impact of nanoparticles on the formation of the alginate shell is not discussed. This interaction should be evaluated to ensure it does not interfere with the shell formation process.

Is the study design appropriate and does the work have academic merit?

Yes

Is the work clearly and accurately presented and does it cite the current literature?

Yes

If applicable, is the statistical analysis and its interpretation appropriate?

Partly

Are all the source data underlying the results available to ensure full reproducibility?

Partly

Are the conclusions drawn adequately supported by the results?

Partly

Are sufficient details of methods and analysis provided to allow replication by others?

Partly

Reviewer Expertise:

Micro/nano robotics

We confirm that we have read this submission and believe that we have an appropriate level of expertise to confirm that it is of an acceptable scientific standard, however we have significant reservations, as outlined above.

# Reviewer report: Reviewer response for version 2

**DOI:** 10.21956/openreseurope.20144.r46297

**Published:** 2024-11-28

**Authors:** Samuel Stealey

**Affiliations:** 1 Saint Louis University, Saint Louis, USA

The manuscript by Roberti et al. demonstrates an attractive option for forming microrobots using only mild conditions and non-organic solvents. Such a system is attractive for potential use for drug delivery applications. Here are some minor comments the authors should address to enhance the discussion of their results:

 1) With the intended end application being drug delivery, can the authors describe how therapeutics may be loaded within the microbots? For example, would these therapeutics be added to hydrogel precursor solutions prior to core-shell formation? If so, could the loaded therapeutic diffuse out throughout and following the subsequent washing/melting/gelation steps? If therapeutics were to be loaded following stable, core-shell formation, have others in the literature achieve sufficient drug loading?

 2) The authors should report the polydispersity in of the measured agarose cores within the manuscript. It is implied that they are quite monodisperse, but with the small sample sizes measured, it is difficult to judge the degree to which they are of similar size.

 3) Could the authors employ rudimentary methods to assess deformability? While advanced and precise techniques such as nano-indentation and flow through capillaries may be outside the scope of this feasibility study, it may be beneficial to manually compress the relatively large spheres and observe the effect on microbot size and structure. For example, a glass slide could be added on top of the large spheres, allowing for imaging during and after deformation. 

 4) The authors should discuss the feasibility of reducing the size of the agarose core beads to physiologically relevant sizes. Can this be achieved using a similar method, or would another method be required?

 5) Following the "melting" of the agarose core, can the authors speak to the relative molecular weight of the agarose polymers? Would these non-gelled polymer strands be expected to diffuse out of the aqueous interior, or would they remain in the core, potentially providing for a crowded environment that could slow diffusion marginally?

Is the study design appropriate and does the work have academic merit?

Yes

Is the work clearly and accurately presented and does it cite the current literature?

Yes

If applicable, is the statistical analysis and its interpretation appropriate?

Yes

Are all the source data underlying the results available to ensure full reproducibility?

Yes

Are the conclusions drawn adequately supported by the results?

Partly

Are sufficient details of methods and analysis provided to allow replication by others?

Yes

Reviewer Expertise:

Hydrogel microspheres, biomaterials testing

I confirm that I have read this submission and believe that I have an appropriate level of expertise to confirm that it is of an acceptable scientific standard, however I have significant reservations, as outlined above.

# Reviewer report: Reviewer response for version 2

**DOI:** 10.21956/openreseurope.20144.r45505

**Published:** 2024-11-06

**Authors:** Mahmut Selman Sakar

**Affiliations:** 1 Ecole Polytechnique Federale de Lausanne, Lausanne, Vaud, Switzerland

The revisions are appropriate. The manuscript can be accepted without further changes.

Is the study design appropriate and does the work have academic merit?

Yes

Is the work clearly and accurately presented and does it cite the current literature?

Yes

If applicable, is the statistical analysis and its interpretation appropriate?

Partly

Are all the source data underlying the results available to ensure full reproducibility?

Yes

Are the conclusions drawn adequately supported by the results?

Yes

Are sufficient details of methods and analysis provided to allow replication by others?

Yes

Reviewer Expertise:

Microrobotics, microfabrication, hydrogels, magnetic actuation

I confirm that I have read this submission and believe that I have an appropriate level of expertise to confirm that it is of an acceptable scientific standard.

# Reviewer report: Reviewer response for version 1

**DOI:** 10.21956/openreseurope.18062.r37273

**Published:** 2024-02-22

**Authors:** Dr. Sudhir G. Warkar

**Affiliations:** 1 Delhi Technological University, Delhi, India

**Comments of Reviewer:**

 In the paper entitled “Fabrication of hydrogel mini-capsules as carrier systems” the authors proposed the synthesis of microrobots made as deformable capsules with hydrogel shells and aqueous cores, having the potential added advantages of biocompatibility, permeability, and stimulus-responsiveness. The process of forming alginate hydrogel mini capsules proceed through the dripping of the mixture of alginate and agarose into a calcium chloride solution to form crosslinked shell of alginate and agarose core. The cross-linked alginate shell is de-crosslinked by dipping the beads in sodium citrate dihydrate solution.

 The scientific quality of the paper is good and the scientific study of the paper is relevant to the academic and scientific community. The paper also has adequate references cited. However, the manuscript needs a few minor changes/clarification on the following observations:

**Reviewer has observed the following issues with the manuscript which the authors must address:**

1. The manuscript requires more clarity for better understanding and repeatability.
2. Section 2.2, after de-crosslinking the alginate template, agarose beads were left behind which were transferred to CaCl _2_ solution of different concentrations to form shells of different thicknesses. Authors should clarify in the paper whether these shells formed on agarose beads are of which material.
3. If both agarose and alginate get crosslinked by Ca ^2+^ then why does only alginate get de-crosslinked on immersing in sodium citrate dihydrate.
4. Section 2.2, a more detailed description of step 5 is required- such as before placing the beads at a temperature of 90-95 ^o^C, agarose was in which physical state (solid/liquid). What exactly happens at 90-95 ^o^C? [In the abstract, authors mentioned ‘Finally, the beads are heated to let the agarose melt and diffuse out, leaving a liquid core’. ]
5. More explanation is required for the loading and release of CaCO _3_ and SiO _2_ in the core.
6. The shell thickness of capsules was measured from microscopy images by measuring the colour intensity-whether the results can be validated by other characterization techniques?
7. The insights need to be incorporated in the paper related to how the hydrogel mini capsules developed as biomedical microrobots can be used for controlled navigation, which could allow them to be guided to hard-to-reach target sites inside the human body.

Is the study design appropriate and does the work have academic merit?

Yes

Is the work clearly and accurately presented and does it cite the current literature?

Partly

If applicable, is the statistical analysis and its interpretation appropriate?

Not applicable

Are all the source data underlying the results available to ensure full reproducibility?

Partly

Are the conclusions drawn adequately supported by the results?

Partly

Are sufficient details of methods and analysis provided to allow replication by others?

Partly

Reviewer Expertise:

Polymeric hydrogels for drug delivery, agriculture, metal ion sensing, and water enrichment applications.

I confirm that I have read this submission and believe that I have an appropriate level of expertise to confirm that it is of an acceptable scientific standard, however I have significant reservations, as outlined above.

# Response

**Published:** 2024-09-24

**Authors:** Elisa Roberti

**Affiliations:** BioRobotics Institute, Sant'Anna School of Advanced Studies, Pisa, Tuscany, Italy

1. Thank you for your feedback. Please consider that in addition to the manuscript, we have uploaded a video demonstrating the protocol. We believe that this provides a clearer visualization and a better understanding, and that it enhances repeatability. In addition, we have addressed all reviewers’ comments to further making our manuscript and process clear, easy to understand and reproduce.
2. The agarose beads are submerged in calcium chloride solutions of varying concentrations to absorb the solution. When the CaCl2-loaded agarose beads are transferred into a fresh alginate solution (containing iron oxide nanoparticles), the calcium ions diffuse out, immediately crosslinking the alginate around the agarose beads, forming the shell. The shell is thus made of a Ca-alginate hydrogel containing iron oxide nanoparticles.
3. It is worth clarifying that only alginate crosslinks due to the presence of calcium ions, while agarose forms a gel via a physical mechanism when cooled from high temperature to room temperature. We indeed use these different hydrogels, exploiting their distinct gelation mechanisms, exactly to be able to de-crosslink them selectively.
4. Agarose remains in a gel/solid phase at room temperature and liquefies at temperatures above 90°C. To enable the liquefication of the agarose gel and the diffusion of agarose molecules through the alginate shell, we heat the core-shell particles (with agarose as the core and alginate as the shell) to 90-95°C, ideally resulting in an agarose-free liquid core surrounded by an alginate shell. However, we have observed that agarose does not fully diffuse out as expected.
5. In microrobots fabrication, it can be useful to load not only magnetic nanoparticles, but also microparticles of other kinds. As an example, we tried these two different kinds of particles, with CaCO _3_ being also sensitive to acidic pH. SiO _2_ microparticles were loaded in the agarose solution before realizing the cores, while CaCO _3_ microparticles were nucleated directly in the already-made cores as an alternative loading method, through the reaction of CaCl _2_ with Na _2_CO _3_. The aim of this experiment was to verify the possibility to load or nucleate particles inside the core, as well as to aid the visualization of the final consistency of the core: we thus dissolved the shell and observed how the loaded particles were released to infer whether the core was fully liquid or had the consistency of a very soft gel.
6. The shell thickness was also measured directly with the measuring tool of our digital microscope, for the capsules whose shell was thick enough to be detected. The colour intensity calculation was performed to also extrapolate data for shells that were not directly measurable by optical microscopy. Our results suggest that there is in fact no substantial/continuous shell for low CaCl _2_ concentrations.
7. *The "Conclusions" section was revised to address the question.*

# Reviewer report: Reviewer response for version 1

**DOI:** 10.21956/openreseurope.18062.r36396

**Published:** 2024-01-23

**Authors:** Mahmut Selman Sakar

**Affiliations:** 1 Ecole Polytechnique Federale de Lausanne, Lausanne, Vaud, Switzerland

This article introduces a technique to fabricate capsules with an alginate shell and liquid core. The technique is described in detail, accompanied by a video that contains step-by-step descriptions. The shell thickness is measured using optical images. Two methods to estimate the shell thickness are proposed: from the CaCl _2_ concentration and from the shell colour intensity. The colour difference is due to the presence of iron oxide nanoparticles. Here are some comments on the rationale behind the design of the capsules and the presentation of the methodology.

 1. Authors hypothesize that the ability to move across tissues and barriers requires "ultradeformability". Please provide a technical description of "ultradeformability". How do we measure the deformability of a liquid-filled soft shell? How does this measure depend on shell thickness, mechanical properties of the shell, and the viscosity of the liquid in the core? What is the role of osmotic pressure?

 2. What is the desired level of deformability? Where is this specification come from (i.e., the size of the holes in the barrier)? Do we know how deformable the immune cells are as a starting point? Can they penetrate into solid tumour? I brought up solid tumour as this is the case mentioned by the authors in the introduction. If the immune cells cannot, would it be reasonable to believe that an engineered capsule can?

 3. As a follow up to the first two comments, what is the liquid inside the capsule? If the liquid inside the capsule is molten, de-polymerized agarose instead of water as intended, would that influence the deformability of the capsule or the release of the cargo? 

 4. Please substantiate the claim that none of the existing fabrication methods are suited to fabricate core-shell capsules without damaging potential payloads. There are numerous articles on the fabrication of hydrogel-containing core–shell capsules, specifically alginate or chitosan as the shell material. Majority of these articles show encapsulation and release of drugs. What am I missing here?

 5. The alginate capsule is permeable and highly deformable. How would the cargo be protected while crossing barriers? How do you envision to implement selective permeability in order to protect the cargo and release at the target location? Is this strategy compatible with the way the capsules are fabricated?

 6. I could not find statistics on how many capsules were fabricated, size range, and dispersity. Why do we see few data points on the plots? Considering the throughput of the fabrication method, I would expect to see many measurements per condition (e.g., CaCl _2_ concentration) with mean and standard deviation instead of a single data point. 

 7. Why is the volume of the alginate shell *V _s_*  proportional to the amount of Ca ^2+^ ions in the core. Please plot shell thickness with respect to capsule size. That would nicely accompany your illustration shown in Figure 2.

 8. What is the effect of adding iron oxide nanoparticles into the alginate to the stiffness of the shell? Is this strategy compatible with the overall objective of making "ultradeformable" capsules?

 9. The discussion does not address an important issue. What future steps will transform the core-shell capsules described in this work into microrobots? How do you envision to implement the required fabrication steps and control strategies? What are the trade-offs and challenges in terms of material choice and design parameters? For example, if magnetic force is going to be applied to pull these capsules through barriers, magnetic volume must be increased which will inherently constrain the deformability of the shell.

Is the study design appropriate and does the work have academic merit?

Yes

Is the work clearly and accurately presented and does it cite the current literature?

Yes

If applicable, is the statistical analysis and its interpretation appropriate?

Partly

Are all the source data underlying the results available to ensure full reproducibility?

Yes

Are the conclusions drawn adequately supported by the results?

Yes

Are sufficient details of methods and analysis provided to allow replication by others?

Yes

Reviewer Expertise:

Microrobotics, microfabrication, hydrogels, magnetic actuation

I confirm that I have read this submission and believe that I have an appropriate level of expertise to confirm that it is of an acceptable scientific standard, however I have significant reservations, as outlined above.

# Response

**Published:** 2024-09-24

**Authors:** Elisa Roberti

**Affiliations:** BioRobotics Institute, Sant'Anna School of Advanced Studies, Pisa, Tuscany, Italy

1. We thank the reviewer for this question, which allows us to better explain the concept of ultra-deformability in the context of micro robotics. In the literature, the term "ultradeformability" is primarily associated with liposomes or transferosomes, particularly those used for transdermal delivery. In that specific scenario, there is a requirement for vesicles capable of substantial squeezing to pass through skin pores without losing their cargo (Souto et al., 2021; Sharma et al., 2024). We broadly define “ultra-deformability” as the ability of microrobots/structures to deform substantially under small external forces (same order of magnitude as those needed to drag them through a liquid) and to pass through openings that are smaller than their size (Vorselen et al., 2020)(Simrah et al., 2024). Possible methods to measure deformability could involve compelling these soft beads to traverse a capillary, nanoindentation, or AFM in force distance curve-based imaging mode. (Vorselen et al., 2020). These techniques are also commonly employed for liposomes or polymersomes. Lowering the thickness and/or elastic modulus of the shell reduces its bending and tensional stiffness, thus increasing the deformability/compliance of the whole structure. The viscosity of the liquid core influences the rate of deformation, as well as the effective deformability of the structure (the lower the viscosity, the lower the forces/stresses required for the liquid to flow within the core). The viscosity can play an additional role if the mechanism of deformation involves a change of volume and flow of the core liquid through the porous shell (the lower the viscosity, the lower the flow resistance). As for the osmotic pressure, its changes can induce swelling or deswelling of the shell and structure, leading to a volume change and change of mechanical properties of the shell. We have thus revised our manuscript to provide an explanation of the concept of ultra-deformability. 
2. Leukocytes are sufficiently deformable and elastic to move inside the small capillaries (diameter < 5 µm) that reach solid tumors, to travel through the interstitial spaces of tissues (< 1 µm), and to extravasate through the nanometric junctions between the endothelial cells of the leaky vasculature in the tumor microenvironment (100 to 500 nm (Wilhelm et al., 2016), depending on the tumor type and stage). Leukocytes can indeed migrate to and infiltrate inside tumors (Wu et al., 2022). If deformable enough, capsules of diameter ≳ 10 µm should be able to at least move in small capillaries. By making them smaller or even more deformable, we could potentially enable their extravasation from blood vessels, particularly considering the gaps between endothelial cells in tumor tissue. Nonetheless, here we report a preliminary investigation of a fabrication method potentially useful for preparing ultra-deformable capsules. The assessment of the fabrication method is performed on millimeter-sized capsules to ease the process and analysis, and further effort would be needed to adapt the method to make microcapsules of the size required for the envisioned applications.
3. Our initial assumption was that once the agarose melted, it would easily diffuse out of the shell, leaving a completely liquid aqueous core. However, we realized that although the agarose melts, the core is not fully liquid (see Extended Data). This could negatively affect the deformability of the capsules. Nonetheless, we have chosen the lowest concentration of agarose that allows the formation of a gel in the initial steps of the process, to minimize the amount of residual agarose in the core after its melting and diffusion out of the shell. With this in mind, we assume that the non-complete liquefication of the core will eventually affect the deformability of the capsule, yet only negligibly. For what concerns the release of the cargo, cargo diffusivity, hydrophilicity and dimensions will have to be taken into account, but we do not expect agarose residues inside the core to make a substantial difference, as deformability and cargo release will mostly depend on the shell properties.
4. We agree that many processes exist to fabricate hydrogel-containing core-shell capsules, e.g. the template-based formation of alginate-chitosan capsules through the layer-by-layer method. However, we have been looking for methods that lead to capsules where both the shell and the core are aqueous, that allow the core to be liquid, and that use only very mild conditions (e.g. ~neutral pH in all steps, to allow the loading of pH-sensitive cargos). None of the reported fabrication methods, to the best of our knowledge, have all the features we need for our purposes (e.g. LbL deposition of chitosan requires acidic pH). Therefore, we have conceived this new fabrication process, and we report here a preliminary assessment of the produced capsules, confident that it could complement the various fabrication processes already reported in the literature.
5. Alginate nano and microparticles have been extensively used to obtain optimal drug delivery systems (Hariyadi and Islam, 2020; He et al., 2020; Abourehab et al., 2022), due to some of the properties of this hydrogel such as the low toxicity, chemical versatility, and pH sensitivity. Notably, there are many examples of alginate-based drug delivery systems capable of targeting and releasing cargos under specific conditions, such as at the low acidic environment near tumors (He et al., 2020). Ideal drug-delivery systems are expected to securely encapsulate drugs without leakage, before reaching the target site and release the drugs upon reaching the target. Alginate-based nanoparticles can accumulate at the target site via passive or active targeting, but some strategies are needed to disassemble the capsule or increase permeability, developing stimuli-responsive capsules. In our specific case, the deformability for crossing the barriers is firstly given by the bending of the shell, and in part by its extension/compression, aiming at minimizing the inner volume changes so as to minimize the exchange of liquids between the core and the environment, decreasing the cargo losses while crossing barriers. Moreover, the volume change can be stimuli-induced and used to trigger the cargo release.
6. We realized many samples, then we thoroughly analyzed from 3 to 5 particles per each experimental condition, and considering the high reproducibility of the results, we assumed it to be sufficient. Then considering the samples number, we thought it was more meaningful to
  report the values of the single samples rather than mean and standard deviation.
7. *The thickness, and thus the volume of the alginate shell is proportional to the amount of Ca ^2+^ ions that were previously loaded into the core because it determines the number of cross-link bonding that each bead can realize. Once the core is put in a solution at a fixed alginate concentration, the Ca ^2+^ ions will be released, inducing the crosslinking of the alginate in the nearby of the cores, and producing a shell whose thickness will be (at first approximation) determined by the number of Ca ^2+^ ions loaded in the core itself (which could be approximated as the product of CaCl _2_ concentration times the volume of the core, dealing with a hydrogel which is mostly* water). Nonetheless, the most important parameter that we are changing here is the CaCl _2_ concentration, whereas there is no large variation among the core radii, with the small polydispersity observed being due to the fabrication method. Moreover, the simple reported model assumes that the core has constant volume during the process, whereas we observed experimentally that the cores undergo a certain degree of swelling and deswelling during the process, also depending on the concentration of CaCl _2_. For these reasons, we have not included the resulting plot of shell thickness versus capsule core radius as it is not informative and shows no clear correlation in the small range of radii observed.
8. The current amount of nanoparticles used is very low, sufficient only for a clear visualization of the capsules but irrelevant from a deformation standpoint. To obtain magnetic microrobots, much higher concentrations of magnetic nanoparticles (or ferrofluid) will be needed. However, by using nanoparticles with a diameter of less than 10 nm and potentially placing them in the core instead of the shell, the impact on deformability will be minimized.
9. *The "Conclusions" section was revised to address the question*.

Abourehab, M.A.S. *et al.* (2022) ‘Alginate as a Promising Biopolymer in Drug Delivery and Wound Healing: A Review of the State-of-the-Art’, *International Journal of Molecular Sciences*, 23(16), p. 9035. Available at: https://doi.org/10.3390/ijms23169035.

 Hariyadi, D.M. and Islam, N. (2020) ‘Current Status of Alginate in Drug Delivery’, *Advances in Pharmacological and Pharmaceutical Sciences*, 2020, p. 8886095. Available at: https://doi.org/10.1155/2020/8886095.

 He, L. *et al.* (2020) ‘Alginate-Based Platforms for Cancer-Targeted Drug Delivery’, *BioMed Research International*, 2020(1), p. 1487259. Available at: https://doi.org/10.1155/2020/1487259.

 Li, T. *et al.* (2018) ‘Cell-based drug delivery systems for biomedical applications’, *Nano Research*, 11(10), pp. 5240–5257. Available at: https://doi.org/10.1007/s12274-018-2179-5.

 Muller, W.A. (2003) ‘Leukocyte–endothelial-cell interactions in leukocyte transmigration and the inflammatory response’, *Trends in Immunology*, 24(6), pp. 326–333. Available at: https://doi.org/10.1016/S1471-4906(03)00117-0.

 Sharma, A. *et al.* (2024) ‘Transfersomes as a Surfactant-based Ultradeformable Liposome’, *BIO Web of Conferences*. Edited by P.K. Prabhakar, 86, p. 01021. Available at: https://doi.org/10.1051/bioconf/20248601021.

 Simrah *et al.* (2024) ‘Transfersome, an ultra-deformable lipid-based drug nanocarrier: an updated review with therapeutic applications’, *Naunyn-Schmiedeberg’s Archives of Pharmacology*, 397(2), pp. 639–673. Available at: https://doi.org/10.1007/s00210-023-02670-8.

 Souto, E.B. *et al.* (2021) ‘Elastic and Ultradeformable Liposomes for Transdermal Delivery of Active Pharmaceutical Ingredients (APIs)’, *International Journal of Molecular Sciences*, 22(18), p. 9743. Available at: https://doi.org/10.3390/ijms22189743.

 Vorselen, D. *et al.* (2020) ‘Mechanical Characterization of Liposomes and Extracellular Vesicles, a Protocol’, *Frontiers in Molecular Biosciences*, 7, p. 139. Available at: https://doi.org/10.3389/fmolb.2020.00139.

 Wilhelm, S. *et al.* (2016) ‘Analysis of nanoparticle delivery to tumours’, *Nature Reviews Materials*, 1(5), pp. 1–12. Available at: https://doi.org/10.1038/natrevmats.2016.14.

 Wu, H. *et al.* (2022) ‘An EPR-Independent extravasation Strategy: Deformable leukocytes as vehicles for improved solid tumor therapy’, *Advanced Drug Delivery Reviews*, 187, p. 114380. Available at: https://doi.org/10.1016/j.addr.2022.114380.
